# Inhibition of Human Monoamine Oxidases A and B by Specialized Metabolites Present in Fresh Common Fruits and Vegetables

**DOI:** 10.3390/plants11030346

**Published:** 2022-01-27

**Authors:** Claudio Marcello Marzo, Sofia Gambini, Stefania Poletti, Francesca Munari, Michael Assfalg, Flavia Guzzo

**Affiliations:** Department of Biotechnology, University of Verona, Strada Le Grazie 15, 37134 Verona, Italy; sofia.gambini@univr.it (S.G.); stefania.poletti@univr.it (S.P.); francesca.munari@univr.it (F.M.); michael.assfalg@univr.it (M.A.)

**Keywords:** monoamine oxidase, phenylpropanoid, D-(−)-Quinic acid, untargeted metabolomics, kiwifruit, polyphenols

## Abstract

Diets rich in fruits and vegetables are associated with better psychological wellbeing and cognitive functions, although it is unclear which molecules and mechanisms are involved. One potential explanation is the inhibition of monoamine oxidases (MAOs), which have been linked to several neurological disorders. The present study investigated the ability of kiwifruit to inhibit MAO-A and MAO-B, refining an in vitro assay to avoid confounding effects. Ultra-performance liquid chromatography/mass spectrometry (UPLC-QTOF) and nuclear magnetic resonance spectroscopy (NMR) were used to select individual kiwifruit metabolites for further analysis. Moreover, extracts of other common fruits and vegetables were screened to identify promising candidate inhibitors. Multiple extracts and compounds inhibited both enzymes, and the selective inhibition of MAO-B by the major kiwifruit specialized metabolite D-(−)-quinic acid was observed. These results suggest that fruits and vegetables contain metabolites that inhibit the activity of MAO-A and -B, offering a potential natural option for the treatment of neurological disorders, in which MAOs are involved.

## 1. Introduction

The beneficial effects of a diet rich in fruits, vegetables, nuts, and vegetable-based ingredients are widely recognized, particularly in the context of cardiovascular disease [1]. More recent studies have linked such diets to mental health, including observational and small intervention studies focusing on the benefits of a so-called Mediterranean diet. These reports revealed a positive impact on cognitive performance [2], depression [3], and psychological wellbeing [4,5]. Randomized controlled registered trials have also produced promising results [6]. Observational and interventional studies on the effect of whole dietetic patterns on brain health, while being powerful tools, cannot determine which fruits and vegetables (and which molecules) are responsible for the positive effects. This would require interventional studies with a single fruit or vegetable, which have been carried out, just to give some examples, with gold kiwifruit [7], orange juice [8], and sour cherry [9]. Kiwifruit (*Actinidia deliciosa*) and gold kiwifruit (*Actinidia chinensis*) are of particular interest, as they have shown antidepressant activity in humans and animals [7,10].

Studies have involved in vitro tests, which can identify molecular targets but cannot provide information about efficacy at the organism level, as well as in vivo tests in animal models and humans, which can demonstrate efficacy at the organism level but generally cannot identify molecular targets. One potential link between fruits and vegetables in diet and brain health is the ability of certain metabolites to inhibit the enzymes monoamine oxidase (MAO) [11]. This flavin-containing enzyme (EC 1.4.3.4) catalyzes the oxidative deamination of primary and some secondary amines, including the catecholamines, serotonin, and trace amines. Humans possess two isozymes of MAO, named MAO-A and -B, both located on the outer membrane of mitochondria [11]. The isozymes were originally defined according to differences in substrate and inhibitor sensitivity: MAO-A has a higher affinity for serotonin and norepinephrine and is sensitive to low concentrations of clorgyline, whereas MAO-B prefers β-phenethylamine as a substrate and is inhibited by low concentrations of deprenyl (selegiline) [12]. MAO-A is predominantly expressed in the placenta, intestine, liver, and lungs, whereas MAO-B is the prevalent form in platelets [13]; both appear to play a protective role by catalyzing the oxidative deamination of biogenic and xenobiotic amines [14]. In the brain, MAO-A is predominantly found in catecholaminergic neurons, whereas MAO-B is more abundant in serotonergic and histaminergic neurons, especially in glial cells [15]. In addition, there are some differences in the distribution of MAOs through rodents and humans, indeed there are more type A than type B enzymes throughout the rat brain; whereas, in the human brain, it is the opposite. Serotonergic neurons, therefore, contain the form of MAO that does not preferentially metabolize 5-hydroxytryptamine [11].

Given the role of MAOs in the metabolism of neuroactive amines, monoamine oxidases inhibitors (MAOIs) have been used extensively in clinical states, such as depression, with the aim to increase dysregulated monoamine levels. Nowadays, MAO-A inhibitors have limited therapeutic use, due to potentially dangerous side effects or drug interactions, while MAO-B inhibitors are still in use as an adjunct therapy in Parkinson’s disease to elevate dopamine levels; there is great interest in the development of alternatives, including natural ones [11].

Numerous plant extracts have been tested for their ability to inhibit MAO, including a large-scale screen that identified five herbs used in traditional medicines, with a higher ability to inhibit MAO-B [16]. The analysis of numerous foods and herbal remedies has revealed a large number of plant metabolites with the ability to inhibit one or both isoforms of MAO [17]. One example is the flavonol quercetin, which inhibits both forms of MAO but shows greater activity against MAO-A [18], as supported by molecular docking studies [19]. Other flavonoids preferentially inhibit MAO-B, such as (+)-catechin, (−)-epicatechin and naringenin [20,21], whereas kaempferol and apigenin are more potent inhibitors of MAO-A [22]. Many investigators have focused on β-carbolines, which are bioactive alkaloids found in hallucinogenic plants, tobacco, and coffee [23]. For example, harman is a selective inhibitor of MAO-A, whereas norharman strongly inhibits both forms of the enzyme [24]. The alkaloid piperine and its related compounds, found in long pepper plants (*Piper longum*), are more potent inhibitors of MAO-B [25]. Anthocyanins, the abundant red and blue pigments found in plants such as cherry and red chicory, can also inhibit MAO, although their rarer aglycosylated counterparts (anthocyanidins) are more potent [26].

In this work, the setup of the MAO inhibition assay was initially performed on kiwifruit, both because of the interesting biological activities it has already demonstrated [10] and because it shares, with other fleshy fruits and vegetables, some features that can potentially interfere with the assay (such as acidic pH, presence of various organic acids, and, for fruits, a very high concentration of sugars) [16]. Thus, we used it as the model fruit. High-resolution techniques (UPLC-MS and NMR) were used to select individual kiwifruit metabolites for assessing their effects on MAO-A and -B. Other juices, namely sweet cherry, apple, nectarine, peach, pear, carrot, fennel, cucumber, lettuce, tomato, red chicory, bell pepper, onion, and shallot, were subsequently screened to evaluate their inhibition activity on MAOs.

The results presented in this study demonstrate the ability of the juices of common fruits and vegetables to reduce MAOs activity in vitro suggesting, if data were replicated in vivo, a possible application as a supplementation tool in the treatment of various neurodegenerative disorders.

## 2. Materials and Methods

### 2.1. Reagents

Clorgyline, R-(−)-deprenyl, human enzymes MAO-A and -B, expressed in baculovirus-infected insect cells (BTI-TN-5B1-4), caffeic acid, (+)-catechin hydrate, Bradford reagent, and bovine serum albumin (BSA), were obtained from MilliporeSigma (Milan, Italy). D-(−)-quinic acid was purchased from Santa Cruz Biotechnology (Heidelberg, Germany). Esculin was obtained from Extrasynthese (Genay, France).

### 2.2. Fruit and Vegetables Sampling

A total of 18 different fruit and vegetable samples were tested, namely apple (*Malus domestica*) cv. golden delicious and cv. prussian, bell pepper (*Capsicum annuum*, ripe and unripe samples), carrot (*Daucus carota*), sweet cherry (*Prunus avium*) cv. black star and cv. roana, cucumber (*Cucumis sativus*), fennel (*Foeniculum vulgare*), kiwifruit (*Actinidia deliciosa* cv. hayward), lettuce (*Lactuca sativa*), nectarine (*Prunus persica* var. *nectarina*), onion (*Allium cepa*), peach (*Prunus persica*), pear (*Pyrus communis*), red chicory (*Cichorium intybus*), shallot (*Allium ascalonicum*), and tomato (*Solanum lycopersicum*). The two different cultivars of apple and sweet cherry were sampled directly in orchards, located in the Veneto Region of Italy; kiwifruits were sourced from local producers, and the other material was bought at the local market in Verona (Italy). To obtain juices with a representative composition, pools of 50 fruits, bulbs (onion and shallot), leaf heads (red chicory and lettuce), or fleshy shoot (fennel) were prepared by cutting slices of each sample, freezing them immediately in liquid nitrogen, and storing them at –80 °C. The frozen material was powdered using an A11 basic analytical mill (IKA-Werke, Staufen, Germany), and the powder was stored at −80 °C.

### 2.3. Fresh Juice Preparation

Each fresh juice was prepared by weighing and thawing 15 g of frozen homogenized powder and centrifuging at 3650× *g* for 15 min at 4 °C. The supernatant was then transferred to a fresh tube and centrifuged at 21,000× *g* for 15 min at 4 °C. The supernatant was passed through a 0.22-µm Millex PES filter (MilliporeSigma, Milan, Italy) and the pH was adjusted to 7.5 with NaOH. For kiwifruit experiments, in order to investigate molecules responsible for the activity on MAOs and limit the number of candidate inhibitors, the fresh juice was passed over a cation exchange cartridge to remove small, polar metabolites. This “neutralized juice” was created by passing the fresh kiwifruit juice through a Discovery DSC-MCAX SPE C8 mixed-mode cation exchange cartridge (MilliporeSigma, Milan, Italy), according to the manufacturer’s instructions, with some minor modifications. Briefly, columns were activated with 15 mL of ethanol for 15 min, then equilibrated with 25 mL of an acid aqueous solution with 1mM of malic acid (pH 3.3) and, finally, samples were loaded. In addition, five “vehicle” solutions were prepared containing the bulk components of fruit juice (various organic acids and/or sugars) (see Results 3.1).

### 2.4. MAO-Glo Assay

The ability of each sample to inhibit MAO in vitro was evaluated in 96-well flat bottom white opaque plates (Thermo Fisher Scientific, Rodano, Italy), using the two-step bioluminescent MAO-Glo assay (Promega, Milan, Italy), following the manufacturer’s instructions. The reaction buffer comprised of 100 mM HEPES (pH 7.5) and 5% (*v*/*v*) glycerol for MAO-A, as well as 10% (*v*/*v*) dimethyl sulfoxide (DMSO) for MAO-B. In each well, 25 µL of the MAO enzyme (20 µg/mL) were mixed with 12.5 µL of the model substrate (40 and 4 µM for MAO-A and -B, respectively) and 12.5 µL of each candidate inhibitor. The final reaction volume was 50 µL. The plate was then incubated at room temperature for 1 h. The luminescent signal was then generated by adding 50 µL of the luciferin detection reagent, incubating the plate for 20 min at room temperature, and measuring the signal using an Infinite 200 Pro microplate reader (Tecan Italia, Cernusco sul Naviglio, Italy). For the blank, the reaction buffer was used, instead of the MAO enzyme. Each experiment also included a positive control with a known selective inhibitor (clorgyline for MAO-A and deprenyl for MAO-B) and negative control with buffer in place of the candidate inhibitor. Based on preliminary assays, fresh juices were tested at selected concentration ranges for each fruit or vegetable (10–700 mg/mL, overall), while concentrations of single compounds ranged from 0.03 mM to 145 mM for selected metabolites (quinic acid, caffeic acid, catechin, and esculin), from 4 nM to 250 nM for clorgyline and 12.5 nM to 2.5 µM for deprenyl.

### 2.5. NMR Spectroscopy

Kiwifruit juice samples for nuclear magnetic resonance (NMR) spectroscopy were prepared by thawing 2–3 g of homogenized powder, vortexing the juice, and sonicating for 15 min in a cold-water bath. The suspension was then centrifuged at 15,000× *g* for 10 min at 4 °C, and the supernatant was transferred to a fresh tube and centrifuged at 18,000× *g* for 20 min at 4 °C to remove the remaining insoluble debris. Then, 0.56 mL of the soluble aqueous extracts was diluted to a final volume of 0.7 mL in 0.15 M potassium phosphate buffer (pH 6.0), 0.02% (*w*/*v*) sodium azide, 5% (*v*/*v*) D_2_O (Cambridge Isotope Laboratories, Cambridge, UK), and 1 mM 4,4-dimethyl-4-silapentane-1-sulfonic acid-d_6_ (DSS-d_6_) (MilliporeSigma, Milan, Italy). NMR spectra were recorded at 298 K using a Bruker Avance III instrument (Bruker, Karlsruhe, Germany), equipped with a triple resonance TCI cryogenic probe and operating at a ^1^H Larmor frequency of 600.13 MHz. The 1H-NOESY spectra were acquired with a mixing time of 100 ms, a recycle delay of 10 s, 64 free induction decays (FIDs), 64,000 data points, and a spectral width of 20 ppm. Spectra were processed with Topspin v3.2 (Bruker) by multiplying FIDs with an exponential weighting function, with line broadening of 0.3 Hz, before Fourier transformation, phasing, and baseline correction. Spectra were then referenced to the DSS-d_6_ singlet signal and analyzed using Chenomx NMR Suite v8.0 (Chenomx, Alberta, Canada) and, by comparison, with the Biological Magnetic Resonance Bank (Retrieved 19 November 2020 from http://www.bmrb.wisc.edu/). The quantity of sugars that are the most abundant metabolites was also determined by NMR analysis of six kiwifruit juice samples. Sucrose and glucose were quantified by integrating the NMR signals using DSS-d_6_ as an internal standard. The Chenomx software was used to quantify fructose. The resulting values were then corrected with the dilution factor and converted to milligrams per 100 grams of homogenized fruit powder, based on the starting weights and volumes (see Section 3.1 and Supplementary File S1).

### 2.6. Protein Concentration Determination

The protein concentration in fresh and neutralized kiwifruit juice was determined using Bradford reagent in a 96-well plate format [27] (see Supplementary File S2). A standard curve was prepared using BSA standards in the concentration range 0.1–1.4 mg/mL. Absorbance was measured on an Infinite 200 Pro microplate reader (Tecan Italia, Cernusco sul Naviglio, Italy).

### 2.7. UPLC-MS Analysis

Fresh and neutralized kiwifruit juices were analyzed through ultra-performance liquid chromatography mass spectrometry (UPLC-MS). The analysis was carried out using an Acquity I Class UPLC system (Waters, Milford, MA, USA), connected to a Xevo G2-XS qTOF mass spectrometer (Waters). All extracts were injected into a Waters ACQUITY UPLC BEH C18 column (2.1 × 100 mm, 1.7 µm), kept at 30 °C, and the mobile phases consisted of 0.1% formic acid in water (A) and acetonitrile (B); the initial conditions were 99% A and 1% B, and the following elution profile was applied: 0–1 min, 1% B; 1–10 min, 1–40% B; 10–13.50 min, 40–70% B; 13.50–15.00 min, 70–90% B; 15.00–16.50 min, 90–100% B; 16.50–20 min, 100% B; 20–20.1 min, 100–1% B (initial conditions). Subsequently, the system was equilibrated in 99% A, and the elution was complete after 25 min. Flow rate was set to 0.35 mL/min. The mass spectrometer was equipped with an electrospray ionization (ESI) source, operating in negative mode. The settings of the ion source were already described in a previous work [28]. In addition, the samples were analyzed with two different MS methods to cover a wider range of *m/z:* for the first method, the quadrupole MS profile was set automatically; for the second method, the profile was set on 300, 400, and 500 *m/z*, with fixed well and ramp time (25% scan time).

The UPLC-MS chromatograms were analyzed using Masslynx v4.1 software (Waters), and MS raw data were processed using Progenesis QI (Nonlinear Dynamics, Newcastle, UK). Metabolite annotations were performed, comparing *m/z* values, isotope distribution, fragmentation (MS/MS), and, where feasible, retention time, with authentic standards, an in-house library of masses built using ProgenesisSDFStudio (Waters), as well as in-house Chemsketch library (ACD Labs, Toronto, ON, Canada) and public libraries, such as mass bank (Retrieved 26 June 2021 from https://massbank.eu/MassBank/Search), Metlin (Retrieved 26 June 2021 from https://metlin.scripps.edu), ChemSpider (Retrieved 26 June 2021 from http://www.chemspider.com).

### 2.8. Quinic Acid Quantification

The calibration curve of D-(−)-quinic acid was prepared by diluting the standard solution at seven different concentrations, for analysis by UPLC-MS, as described above. Linearity was 0.9993, over the quinic concentration range of 0.5–4 μg/mL, derived using the least squares regression analysis. Three replicate samples were used for quantification. In order to overcome possible matrix effects, due to ESI source, various dilutions of the sample were prepared, and spiking experiments, as described by Toffali et al. [29], were used to determine the presence/absence of matrix effects for each dilution. The quantification was performed at the dilution of 1/1600, where the matrix effect was completely absent.

### 2.9. Data Analysis

Statistical analysis was carried out using Prism v9.0 (GraphPad Software, San Diego, CA, USA). Significant differences between samples were determined by one-way analysis of variance (ANOVA), followed by Dunnett’s or Tukey’s post hoc tests. IC_50_ values were calculated by nonlinear regression.

## 3. Results

### 3.1. Kiwifruit as a Model Fruit for MAO Inhibition Assay Optimization and Activity

**Figure 1:**
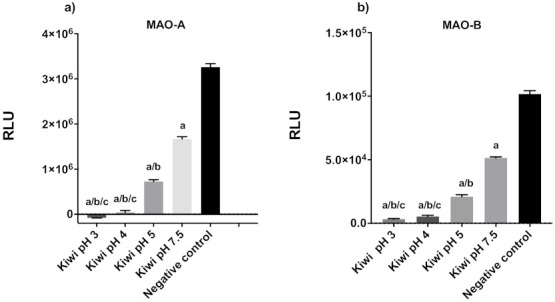
Effects of pH on MAOs activity. MAO-Glo assay of kiwifruit juice (at different pH values) to test for the inhibition of human MAO-A (**a**) and -B (**b**). A total of 12.5 µL of kiwifruit juice, corresponding to 16.8 mg of fresh fruit, was used. Activity was measured in relative light units (RLUs). Values were evaluated by one-way ANOVA, followed by Tukey’s test, and expressed as means ± SD (*n* = 4 per group); ^a^ *p* ≤ 0.0001 vs. negative control group, ^b^ *p* ≤ 0.0001 vs. pH 7.5, ^c^ *p* ≤ 0.0001 vs. pH 5.

Microplate assays to test the ability of compounds to inhibit MAO-A and -B were optimized for use with fruit and vegetable extracts by testing freshly extracted kiwifruit juice. Freshly extracted fruit and vegetable juice is acidic (pH ~3), due to the abundance of organic acids (mainly citric and malic acids), ascorbic acid, and sugars. To evaluate whether pH was an interfering factor in the assay, the pH of the fresh juice was adjusted to 4, 5, and 7.5 and tested its ability to inhibit MAO-A and -B. Low pH strongly inhibited MAO activity [F_(4,10)_ = 2424; *p* < 0.0001], so all the subsequent experiments were adjusted to pH 7.5, the same pH of the assay buffer, to ensure that inhibitory effects were directly caused by the metabolites, rather than the low pH (Figure 1).

**Figure 2:**
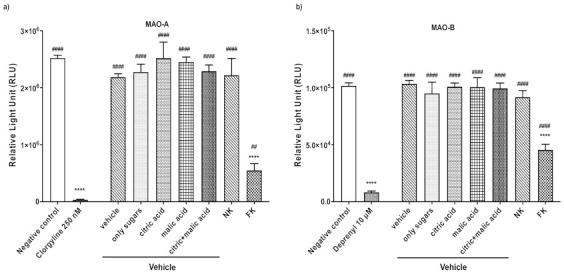
Effects of fresh and neutralized kiwifruit juice and matching vehicle solutions on MAO activity. A total of 12.5 µL of kiwifruit juice, corresponding to 16.8 mg of fresh fruit, was used and equivalent amounts of the other samples. The samples were tested for their ability to inhibit (**a**) MAO-A and (**b**) -B activity using the bioluminescent MAO-Glo assay, with values expressed as relative light units (RLUs), compared to 250 nM clorgyline and 2.5 μM deprenyl as positive controls for MAO-A and -B, respectively, and a negative control with buffer in place of the inhibitor. Data were evaluated by one-way ANOVA, followed by Tukey’s test, and expressed as means ± SD (*n* = 4 per group) **** *p* ≤ 0.0001 vs. negative control group, ^####^ *p* ≤ 0.0001 & ^##^ *p* ≤ 0.01 vs. positive control group (clorgyline/deprenyl). NK = neutralized kiwifruit, FK = fresh kiwifruit.

To exclude the possibility that bulk primary metabolites (such as sugars and citric and malic acid) and ascorbic acid were responsible for the inhibitory activity, vehicle solutions were created using these major components, detected in kiwifruit by NMR spectroscopy (Supplementary File S1) and reported in scientific literature. The composition of the complete vehicle solution was 10 g/L sucrose, 33 g/L fructose, 36 g/L glucose, 0.7 g/L ascorbic acid, 9 g/L citric acid, and 9 g/L malic acid. Four additional vehicle solutions were produced, containing, respectively, only the sugars, citric acid, and/or malic acid. The inhibitory activity of the complete and partial vehicle solutions against the fresh (FK) and neutralized juice (FK), all adjusted to pH 7.5, were then tested. Only the fresh juice showed significant activity, with greater activity against MAO-A than MAO-B (Figure 2).

**Figure 3:**
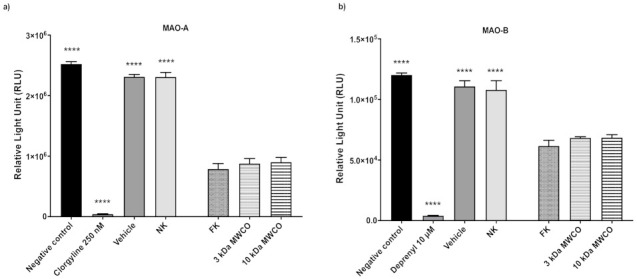
Effect of fresh and protein-depleted kiwifruit juice on MAO activity. A total of 12.5 µL of fresh or protein-depleted kiwifruit juice, corresponding to 16.8 mg of fresh fruit, was used. The samples were tested for their ability to inhibit (**a**) MAO-A and (**b**) -B activity using the bioluminescent MAO-Glo assay, with values expressed as relative light units (RLUs), compared to 250 nM clorgyline and 2.5 μM deprenyl as positive controls for MAO-A and -B, respectively, and a negative control with buffer in place of the inhibitor. Data were evaluated by one-way ANOVA, followed by Dunnett’s test, and expressed as means ± SD (*n* = 4 per group; **** *p* ≤ 0.0001 vs. fresh kiwifruit juice group); MWCO = molecular weight cut-off; NK = neutralized kiwifruit; FK = fresh kiwifruit.

Then, the role of fruit proteins in the observed inhibitory effect was evaluated, since kiwifruits contain proteolytic enzymes, such as actinidin [30]. The protein concentration in kiwifruit (active) and neutralized juice (inactive) was found to be the same, suggesting that proteins are not responsible for the inhibition of MAO. The protein concentration in the fresh juice was 0.548 ± 0.099 mg/mL and that in the neutralized juice was 0.573 ± 0.215 mg/mL (Table S1 and Figure S1). To confirm that kiwifruit proteins do not inhibit MAO, a protein-free derivative of the fresh juice was prepared by passing it through filters, with molecular weight cut-off (MWCO) values of 3 and 10 kDa, before repeating the inhibition assays. As expected, there were no significant differences between the fresh juice and two protein-depleted filtrates, in terms of their ability to inhibit MAO-A and -B (Figure 3).

Having excluded proteins, sugars, organic acids, and ascorbic acid as the source of the inhibitory activity, a list of metabolites present in the active fresh juice, but not the inactive neutralized juice, was prepared, based on the heat map from the untargeted metabolomic analysis (Supplementary File S3). The list of putatively identified, specialized metabolites that were more abundant in the fresh juice included caffeic acid hexoses and di-hexoses, other caffeic acid derivatives, esculin, catechin, quinic acid, and quinic acid derivatives.

The effect of different concentrations of kiwifruit juice was compared to clorgyline and deprenyl standards in the MAO inhibition assay, revealing that the juice has greater activity against MAO-A and lower against MAO-B (Table 1). A list of metabolites that were promising candidate inhibitors was prepared, according to their abundance in fresh kiwifruit juice or a high ratio between fresh and neutralized kiwifruit juice (see Supplementary File S3). Based on these criteria, caffeic acid, quinic acid, catechin, and esculin were selected for further experiments. Since UPLC-MS data showed that quinic acid was the most abundant candidate, and its concentration (491.26 ± 1.80 mg/100 g fruit fresh weight) was also determined by mass spectrometry (see Section 2.8). The effects of caffeic acid, quinic acid, catechin, and esculin standards were compared to clorgyline and deprenyl standards in the MAO inhibition assay (Table 1). It was found that catechin and esculin inhibited both enzymes similarly, caffeic acid inhibited both enzymes but was more active against MAO-B, whereas quinic acid showed selective activity against MAO-B.

### 3.2. Common Fruits and Vegetables Inhibit Human MAO-A and -B

Having optimized the MAO inhibition assays, using kiwifruit as the model, 17 additional samples, representing common fruits and vegetables were tested, using clorgyline and deprenyl as controls. Each sample was tested at different concentrations to establish the IC_50_, as shown in Table 2. MAO-A was most strongly inhibited by extracts of red chicory, followed by cucumber and fennel. In contrast, MAO-B was most strongly inhibited by extracts of cucumber, followed by red chicory and ripe bell pepper.

The chromatographic profiles of the above samples were explored by UPLC-ESI-MS, as shown in Table 3 and Supplementary File S1. The main peaks were tentatively annotated.

## 4. Discussion

In search of possible molecular targets of fresh fruit and vegetables activity on brain health, extracts from 14 species were screened for their ability to inhibit the human enzymes MAO-A and -B in vitro.

The knowledge of the ability of edible fruits and vegetables to inhibit MAO-A and B enzymes has a double meaning. On one hand, the inhibition of the MAOs through diet could contribute to the control of pathologies dependent on the activity of these enzymes. On the other hand, knowledge of the biological activities of diet components is necessary to design the dietary restrictions when MAOs inhibitor drugs are taken (e.g., the cheese reaction in patients under MAOIs treatment could lead to a hypertensive crisis after eating tyramine-rich foods) [31]. Indeed, biogenic amines are abundantly contained in processed foods; however, even fresh fruits and vegetables can contain them, albeit in much smaller quantities [32].

Fruit and vegetable tissues are complex mixtures of insoluble macromolecules (e.g., polysaccharides, structural proteins, lignin, etc.), lipophilic molecules, soluble proteins and carbohydrates, and a complex array of small-molecule metabolites, many with bioactive properties [33]. The insoluble components comprise of most of the dietary fiber, which is largely removed during the juice preparation process (see Section 2.3); whereas the soluble portion still retains a complex mixture of molecules, including proteins, abundant primary metabolites (e.g., sugars, amino acids, and organic acids), and specialized secondary metabolites in various classes, some of which are at low level [34]. In principle, all these components could inhibit MAO-A and/or -B. Moreover, fruits and vegetables are acidic due to the high content of organic acids in the vacuolar sap [35], with citric and malic acids being predominant in many fleshy fruits such as kiwifruit [36]. The effect of kiwifruit pH on the in vitro MAOs activity assay was evaluated, revealing that both enzymes are inhibited per se by the low pH of fruit juice, with a decrease of inhibition as the pH approaches neutrality. The issue of pH was already addressed by Mazzio and colleagues [16], while others have neglected this aspect hence their data may include false positive results. In addition, since fruits such as kiwifruit, fig, papaya and pineapple contain proteolytic enzymes that could damage MAOs, it was also necessary to exclude the possibility that proteases present in the fruit extracts were responsible for MAO inhibition. The assays showed that the protein-free juice adjusted to pH 7.5 retained the ability to inhibit both enzymes, confirming that proteases were not responsible for the inhibitory activity observed in vitro for kiwifruit. Moreover, the use of “neutralized juice” devoid of many small, polar metabolites and ‘vehicle solutions’ pointed at a group of specialized metabolites as possible responsible for the inhibitory activity. The comparison of fresh and neutralized juices allowed the selection of a list of candidate specialized metabolites most likely to inhibit MAO-A and -B, specifically those present in large quantities and those exclusively present in the fresh juice (see Supplementary File S3). This resolved the list of candidates to caffeic acid glucosyl derivatives, catechin, esculin and quinic acid. When tested individually, caffeic acid, catechin and esculin showed non-selective inhibitory activity against both MAOs, whereas quinic acid showed selective inhibitory activity against MAO-B. Caffeic acid, caffeic acid derivatives and catechin (all representing the phenylpropanoid family) have already been reported for their ability to inhibit MAO, but this is the first report of the inhibitory activity of esculin [21,37]. For quinic acid, one previous study described the ability of this molecule to protect rats from aluminum chloride-induced dementia and suggested non-selective inhibition of MAO as a potential mechanism [38]. The selectivity of caffeoylquinic acid derivatives is unclear: two previous articles [37,39] provide evidence for selective activity against MAO-B, whereas another [40] also showed an effect against MAO-A.

According to the results of this work the most interesting specialized metabolite is quinic acid which is mainly and abundantly found in plants as an ester with other secondary metabolites, especially hydroxycinnamic acids such as caffeic acid, and in these forms it is widespread within the plant kingdom [41]. Free quinic acid is usually found at low levels (<100 mg/100 g fresh weight) in fruits and vegetables and edible fruits with high levels of quinic acid are rare [42], since ripe fleshy fruits generally accumulate citric and malic acids as major organic acids. Exceptions include black chokeberry (*Aronia melanocarpa*) [43], Ponkan mandarin (*Citrus reticulata*) and kiwifruit [44]. Free quinic acid is also found in some processed foods due to ester hydrolysis, for example during the roasting of coffee beans [45]. In our analysis, we found that the relative amount of free quinic acid is generally higher in fruits compared with the other plant organs, as shown in Supplementary File S1.

In addition, the in vivo bioavailability of pure quinic acid is not reported in the literature, but its recovery in human serum after coffee consumption suggests it can reach the blood [46] and high levels of quinic acid were detected in ileostomy fluids after the consumption of an apple smoothie (~70%) [47]. The level of quinic acid in the fresh kiwifruit juice was ~492 mg/100 g of fresh fruit, in line with that reported in the literature (390 mg/100 g fresh weight [48]). The high level of quinic acid in kiwifruits and the potential bioavailability of this compound suggest that the promising in vitro activity may be replicated in vivo, and this should be now addressed in further studies. It should be noticed that all polyphenols, including catechin, have poor bioavailability and a limited ability to cross the blood–brain barrier, which hinders their clinical development [49]. However, nanotechnology could help to overcome this challenge by improving penetration and absorption [50].

If the evaluation of the possible MAO-A and -B inhibiting activity by the commonly consumed fresh fruits is poorly known, the activity of fresh vegetable is completely unknown. Thus, different tissues of common vegetable crops, including unripe fruits (cucumber, green bell pepper), ripe fruits (bell pepper, tomato), leaves (red chicory, lettuce), roots (carrot), shoots (fennel), and bulbs (onion, shallot) were tested. All of them showed some ability to inhibit MAO-A and -B in vitro.

The inhibition of MAO-A and/or -B has been extensively reported for extracts of many medicinal plants, including *Artemisia* (IC_50_ MAO-B < 0.7 mg/mL), *Perilla* (IC_50_ MAO-B < 0.2 mg/mL), *Glycyrrhiza* species (IC_50_ MAO-B < 0.07 mg/mL), *Psoralea corylifolia* seeds (IC_50_ MAO-B = 0.054 mg/mL), *Hypericum afrum* (IC_50_ MAO-A = 3.37 μg/mL and IC_50_ MAO-B = 13.50 μg/mL), and, of course, *Banisteriopsis caapi* (IC_50_ MAO-A ∼0.01–0.4 μg/mL), one of the ingredients of the hallucinogenic brew Ayahuasca [16,51,52]. Although fruits and vegetables generally inhibit MAO-A and -B to a lesser degree than herbs and medicinal plants, they are consumed in much greater quantities in the diet.

Few reports have been published on this topic, and most considered extracts of dried, raw material, rather than fresh tissue or used extraction methods involving solvents. These issues hinder comparison with the results of this work because the juices in this study were obtained from nitrogen-frozen fresh material, in order to use raw material as similar as possible to fresh produce consumed in the diet.

Another issue regards the inhibiting power of the individual compounds tested, since in this work MAO-A and -B inhibition was exerted at millimolar (from 0.4 to 34.73) concentration range (see Table 1). It cannot be excluded that the inhibition of the whole extract could depend on the small additive/synergistic contributions of many metabolites, without a predominant role of specific individual compounds. These mechanisms are often overlooked, since the typical research approach is devoted at reducing complexity to identify single active compounds. This explains, in general, why single molecules can’t replace the natural phytocomplexes of fruits and vegetables to achieve the health benefits [53].

This aspect also could be addressed in future studies analyzing the single active components and how their combination affects the activity.

## Figures and Tables

**Table 1:** IC_50_ values of kiwifruit and its secondary metabolites.

Compound	IC_50_ Value			
	MAO-A ^a^		MAO-B ^a^	
	(mg/mL) ^b^	M ^c^	(mg/mL) ^b^	M ^c^
Clorgyline (MAO-A control)	5.49 × 10^−6^ ± 2.4 × 10^−7^	0.0178 ± 0.0007 µM	-	-
Deprenyl (MAO-B control)	-	-	1.96 × 10^−5^ ± 8.2 × 10^−7^	0.1048 ± 0.004 µM
Kiwifruit ^b^	86 ± 2.6	-	122.9 ± 5.5	-
D-(−)-Quinic acid	-	-	6.60 ± 0.25	34.37 ± 1.307 mM
Caffeic acid	0.49 ± 0.02	2.747 ± 0.126 mM	0.08 ± 0.005	0.439 ± 0.03 mM
Catechin	1.88 ± 0.04	6.464 ± 0.121 mM	1.17 ± 0.04	4.029 ± 0.122 mM
Esculin	3.93 ± 0.15	11.55 ± 0.4305 mM	4.10 ± 0.13	12.06 ± 0.3721 mM

The ability of kiwifruit juice, quinic acid, caffeic acid, catechin, and esculin to inhibit MAO-A and -B was determined using the MAO-Glo assay, compared to clorgyline and R-(−)-deprenyl as positive controls for MAO-A and -B, respectively. ^a^ Each inhibitory activity is expressed as the mean of 50% inhibitory concentration (IC_50_) of quadruplicate determinations, obtained by interpolation of the concentration-inhibition curves. ^b^ mg fruit fresh weight/mL. ^c^ For standards metabolite, data are also shown in molarity (M) for comparative purposes.

**Table 2:** IC_50_ values of common fruit and vegetable extracts against MAO-A and -B.

Sample or Compound Tested	IC_50_ Values (mg/mL) ^a^	
	MAO-A ^b^	MAO-B ^b^
Clorgyline	5.49 × 10^−6^ ± 2.4 × 10^−7^	-
R-(−)-Deprenyl	-	1.96 × 10^−5^ ± 8.2 × 10^−7^
Kiwifruit (*Actinidia deliciosa*)	86.0 ± 2.6	122.9 ± 5.5
Apple (*Malus domestica*) cv. golden delicious	186.5 ± 5.9	ND
Apple (*Malus domestica*) cv. prussian	159.0 ± 5.2	100.7 ± 5.3
Carrot (*Daucus carota*)	79.7 ± 4.0	61.5 ± 1.7
Cherry (*Prunus avium*) cv. black star	79.0 ± 1.9	84.9 ± 2.7
Cherry (*Prunus avium*) cv. roana	52.5 ± 0.8	56.3 ± 2.0
Cucumber (*Cucumis sativus*)	15.7 ± 0.5	20.8 ± 0.7
Fennel (*Foeniculum vulgare*)	47.1 ± 2.4	66.7 ± 1.1
Lettuce (*Lactuca sativa*)	69.0 ± 2.4	69.8 ± 1.9
Nectarine (*Prunus persica*)	133.9 ± 4.7	155.5 ± 7.0
Onion (*Allium cepa*)	93.0 ± 3.7	113.7 ± 3.4
Peach (*Prunus persica*)	103.7 ± 2.7	98.3 ± 5.0
Pear (*Pyrus communis*)	106.3 ± 3.0	97.0 ± 3.0
Red chicory (*Cichorium intybus*)	13.8 ± 0.3	29.3 ± 0.6
Ripe bell pepper (*Capsicum annuum*)	59.0 ± 2.7	38.7 ± 1.9
Shallot (*Allium ascalonicum*)	48.2 ± 1.9	47.8 ± 1.7
Tomato (*Solanum lycopersicum*)	81.0 ± 2.8	103.5 ± 3.0
Unripe bell pepper (*Capsicum annuum*)	74.8 ± 4.4	43.6 ± 3.3

The ability of common fruit and vegetable extracts to inhibit MAO-A and -B was determined using the MAO-Glo assay, compared to clorgyline and deprenyl as positive controls for MAO-A and -B, respectively. ND = not determined. ^a^ Inhibitory activity of fruits/vegetables is expressed as mg fresh weight/mL. ^b^ Each inhibitory activity is expressed as the mean of 50% inhibitory concentration (IC_50_ in mg/mL) of quadruplicate determinations, obtained by interpolation of the concentration-inhibition curves.

**Table 3:** Metabolites annotated and their occurrence in the fruits and vegetables under investigation.

Species	Id	Rt (min)	*m/z* (-) Detected	*m/z* (-) Expected	Elemental Formula	Putative Identification	Fragments	Main Adduct
DC, FV, AD, LS, CI, MDG, MDP, PC, PPN, PP, RCA, UCA, SL	1	0.80	533.174	533.171	C_18_H_32_O_15_	glucose–glucose–rhamnose		fa
DC, AC, AA, FV, AD, LS, CI, MDG, MDP, PC, PPN, PP, RCA, UCA, SL	2	0.80	387.115	387.113	C_12_H_22_O_11_	sucrose		fa
DC, CS, FV, CI, MDP, PP, UCA	3	0.75	96.961	96.969	H_3_PO_4_	phosphate		M-1H^+^
DC, CS, PAR, FV, AD, LS, CI, MDG, MDP, PC, PPN, PP, SL	4	0.86	133.014	133.013	C_4_H_6_O_5_	malic acid	115.003	M-1H^+^
DC, CS, AC, AA, FV, AD, LS, CI, MDG, MDP, PPN, PP, RCA, UCA, SL	5	1.32	191.020	191.019	C_6_H_8_O_7_	citric acid	111.008	M-1H^+^
DC	6	4.97	431.120	431.118	C_17_H_22_O_10_	sinapic acid hexoside	222.993	fa
CS	7	3.45	315.072	315.071	C_13_H_16_O_9_	dihydroxybenzoic acid hexoside	150.016	M-1H^+^
PAB, PAR, PPN, PP	8	4.00	353.088	353.087	C_16_H_18_O_9_	caffeoyl quinic acid	135.034; 179.034	M-1H^+^
PAB, PAR	9	4.58	337.093	337.092	C_16_H_18_O_8_	coumaroyl quinic acid	163	M-1H^+^
PAB, PAR	10	4.66	477.161	477.16	C_19_H_28_O_11_	caffeoyl quinic acid derivative	135.034; 179.034; 353.087	fa
PAB, PAR, SL	11	6.26	609.146	609.145	C_27_H_30_O_16_	quercetin-O-rutinoside	300.027	M-1H^+^
PAR	12	4.72	593.151	593.15	C_27_H_31_O_15_	cyanidin-O-rutinoside	284.032	M-1H^+^
PAR, AD, LS, CI, MDG, MDP, PC, PPN, PP, RCA, UCA, SL	13	0.79	191.056	191.055	C_7_H_12_O_6_	quinic acid	127.045	M-1H^+^
AC, AA	14	1.87	873.273	873.272	C_30_H_52_O_26_	penta-hexose	827.268	fa
AC, AA	15	2.22	873.273	873.272	C_30_H_52_O_26_	penta-hexose	827.268	fa
AC, AA, LS	16	2.47	1035.327	1035.325	C_36_H_62_O_31_	hexa-hexose	989.322	fa
AC, AA	17	2.54	1197.379	1197.377	C_42_H_72_O_36_	hepta-hexose	1151.374	fa
AC, AA	18	5.64	625.141	625.14	C_27_H_30_O_17_	quercetin-O-dihexoside	301.034; 464.087	M-1H^+^
AC, AA	19	5.83	639.156	639.156	C_28_H_32_O_17_	rhamnetin/isorhamnetin-O-dihexoside	313.034; 315.049	M-1H^+^
AC, AA	20	7.23	463.088	463.087	C_21_H_20_O_12_	quercetin-O-hexoside	151.002; 178.997; 301.034	M-1H^+^
AC, AA	21	7.56	477.103	477.103	C_22_H_22_O_12_	rhamnetin/isorhamnetin-O-hexoside	314.042	M-1H^+^
AC, AA, LS, CI	22	0.82	549.166	549.166	C_18_H_32_O_16_	tri-hexose		fa
AA	23	1.11	711.221	711.219	C_24_H_42_O_21_	tetra-hexose		fa
CI	24	6.58	461.072	461.072	C_21_H_18_O_12_	kaempferol-O-glucuronide	285.039	M-1H^+^
AA	25	2.66	1359.432	1359.43	C_48_H_82_O_41_	octo-hexose	1313.427	fa
DC, PP	26	3.52	329.087	329.087	C_14_H_18_O_9_	vanillic acid glucoside	108.021; 123.046; 152.011; 167.034	
DC	27	3.62	465.125	465.103	C_21_H_22_O_12_	taxifolin-O-hexoside		M-1H^+^
FV	28	7.06	429.140	429.118	C_22_H_22_O_9_	hydroxy methoxy flavone-O-hexoside		M-1H^+^
FV	29	5.70	367.103	367.102	C_17_H_20_O_9_	feruloyl quinic acid	193.05	M-1H^+^
CS, FV, LS	30	2.51	312.095	312.094	C_10_H_13_N_5_O_4_	adenosine	134.046	fa
FV	31	2.67	282.083	282.083	C_10_H_13_N_5_O_5_	guanosine	133.015; 150.04	M-1H^+^
AD	32	3.99	341.088	341.087	C_15_H_18_O_9_	caffeic acid glucoside	135.034; 179.034	M-1H^+^
AD	33	4.59	341.088	341.087	C_15_H_18_O_9_	caffeic acid glucoside	135.034; 179.034	M-1H^+^
AD	34	4.25	339.071	339.071	C_15_H_16_O_9_	esculin	137.024; 177.018	M-1H+
AC, AA	35	0.78	176.038	176.038	C_6_H_11_NO_3_S	alliin/isoalliin		M-1H^+^
AC	36	0.73	150.022	150.022	C_4_H_9_NO_3_S	methiin		M-1H^+^
LS	37	10.40	329.232	329.232	C_18_H_34_O_5_	trihydroxy-octadecenoic acid (oxylipin)		M-1H^+^
LS	38	9.82	327.217	327.217	C_18_H_32_O_5_	trihydroxy-octadecadienoic acid (oxylipin)		M-1H^+^
LS, CI	39	1.86	873.274	873.272	C_30_H_52_O_26_	penta-hexose	827.268	fa
LS	40	1.79	243.061	243.061	C_9_H_12_N_2_O_6_	uridine		M-1H^+^
MDG, MDP, PC, PPN, PP	41	4.68	353.088	353.087	C_16_H_18_O_9_	chlorogenic acid	135.034; 179.034; 191.055	M-1H^+^
MDG, MDP	42	5.03	577.135	577.134	C_30_H_26_O_12_	procyanidin P2 type	289	M-1H^+^
MDG, MDP	43	5.45	337.092	337.092	C_16_H_18_O_8_	coumaroyl quinic acid	163.039; 173.045; 119.049	M-1H^+^
MDG, MDP, PC	44	5.35	289.071	289.071	C_15_H_14_O_6_	epicatechin		M-1H^+^
MDP	45	5.53	865.197	865.197	C_45_H_38_O_18_	procyanidin P3 type		M-1H^+^
MDG, MDP	46	7.12	567.173	567.171	C_26_H_32_O_14_	phloretin 2′-O-xylosyl glucoside	167.035; 273.075	M-1H^+^
MDG, MDP	47	7.05	447.092	447.092	C_21_H_20_O_11_	quercetin-O-desoxyhexoside		M-1H^+^
MDG, MDP	48	7.21	567.173	567.171	C_26_H_32_O_14_	phloretin-O-xylosyl glucoside structural isomer		M-1H^+^
MDG, MDP	49	7.67	435.129	435.129	C_21_H_24_O_10_	phloretin-O-glucoside		M-1H^+^
MDG, MDP	50	4.85	353.089	353.087	C_16_H_18_O_9_	caffeoyl quinic acid	179.034; 191.055	M-1H^+^
PPN, PP	51	4.34	577.134	577.134	C_30_H_26_O_12_	procyanidin P2 type	289	M-1H^+^
PP	52	4.71	289.071	289.071	C_15_H_14_O_6_	catechin		M-1H^+^
PC	53	3.49	365.134	365.134	C_17_H_22_N_2_O_7_	tryptophan glucose	203.081	M-1H^+^
PC	54	3.88	447.117	447.113	C18H24O13	dihydroxybenzoic acid hexose pentose	153.018	M-1H^+^
PC	55	3.97	447.117	447.113	C_18_H_24_O_13_	dihydroxybenzoic acid hexose pentose	153.018	M-1H^+^
PC	56	6.77	623.162	623.161	C_28_H_32_O_16_	isorhamnetin-O-galattosyl rhamnoside		M-1H^+^
PC	57	6.83	623.162	623.161	C_28_H_32_O_16_	isorhamnetin-O-rutinoside		M-1H^+^
PC	58	7.00	477.103	477.103	C_22_H_22_O_12_	isorhamnetin-3-O-galactoside	314.0425	M-1H^+^
PC	59	7.09	477.103	477.103	C_22_H_22_O_12_	isorhamnetin-3-O-glucoside	314.0425	M-1H^+^
PC	60	7.40	519.114	519.113	C_24_H_24_O_13_	isorhamnetin-3-O-acetyl galactoside		M-1H^+^
PC	61	7.51	519.114	519.113	C_24_H_24_O_13_	isorhamnetin-3-O-acetyl glucoside		M-1H^+^
SL	62	8.19	1127.548	1127.548	C_51_H_86_O_24_	tomatoside A	1081.875; 919.491	fa
SL	63	6.73	1314.597	1314.596	C_58_H_95_NO_29_	esculeoside A	1268.591; 1136.548	fa
RCA, UCA	64	9.57	1129.528	1129.527	C_50_H_83_O_25_	triterpenoid saponin	1083.524; 921.470	fa
CI	65	6.46	477.067	477.066	C_21_H_18_O_13_	quercetin-O-glucuronide		M-1H^+^
CI	66	6.79	505.096	505.098	C_13_H_22_O_13_	querceti-O-acetyl-glucoside	301.035	M-1H^+^
CI	67	7.02	339.054	339.053	C_15_H_16_O_7_S	deoxylactucin sulphate		M-1H^+^
CI	68	7.41	259.097	259.097	C_15_H_16_O_4_	8-deoxylactucin	215.106	M-1H^+^
DC, CS, AC, AA, FV, AD, LS, CI, RCA, UCA	69	0.72	145.061	145.061	C_5_H_10_N_2_O_3_	glutamine		M-1H^+^
DC, CS, AA, CI, UCA, SL	70	0.72	132.030	132.029	C_4_H_7_NO_4_	aspartic acid		M-1H^+^
CS	71	5.43	901.241	901.24	C_42_H_46_O_22_	kaempferol-O-(coumaroyl) glucoside rutinoside	739.186	M-1H^+^
PAB, PAR, MDG, MDP, PC, PPN, PP	72	0.78	181.071	181.071	C_6_H_14_O_6_	sorbitol		M-1H^+^
DC, PAB, PAR, FV, AD, PPN, SL	73	0.74	195.050	195.05	C_6_H_12_O_7_	gluconic acid		M-1H^+^
RCA, UCA	74	0.80	175.024	175.024	C_6_H_8_O_6_	ascorbic acid		M-1H^+^
RCA, UCA, SL	75	1.05	175.024	175.024	C_6_H_8_O_6_	ascorbic acid		M-1H^+^
AD, MDG, SL	76	0.73	146.042	146.45	C_5_H_8_NO_4_	glutamic acid		M-1H^+^
DC, PAB, PAR, CI, MDG, PC, PPN, RCA, UCA	77	0.73	131.045	131.045	C_4_H_8_N_2_O_3_	asparagine		M-1H^+^
PAB, PAR	78	0.77	343.124	343.124	C_12_H_24_O_11_	sorbitol–glucose	181.069	M-1H^+^

The ids are the same of Supplementary File S1. List of abbreviations: AA = shallot (*Allium ascalonicum*), AC = onion (*Allium cepa*), AD = kiwifruit (*Actinidia deliciosa*), CI = red chicory (*Cichorium intybus*), CS = cucumber (*Cucumis sativus*), DC = carrot (*Daucus carota*), FV = fennel (*Foeniculum vulgare*), LS = lettuce (*Lactuca sativa*), MDG = apple (*Malus domestica*) cv. golden delicious, MDP = apple (*Malus domestica*) cv. prussian, PAB = cherry (*Prunus avium*) cv. black star, PAR = cherry (*Prunus avium*) cv. roana, PC = pear (*Pyrus communis*), PP = peach (*Prunus persica*), PPN = nectarine (*Prunus persica*), RCA = ripe bell pepper (*Capsicum annuum*), SL = tomato (*Solanum lycopersicum*), UCA = unripe bell pepper (*Capsicum annuum*); Fa = formic adduct.

## Data Availability

Not applicable.

## Supplementary Materials

The following supporting information can be downloaded at: https://www.mdpi.com/article/10.3390/plants11030346/s1, File S1: Chromatographic profiles; File S2: Bradford Assay; File S3: Untargeted metabolomics data matrix.
