# Supplementary material for: Inhibition of Human Monoamine Oxidases A and B by Specialized Metabolites Present in Fresh Common Fruits and Vegetables
Source: Plants (Basel). 2022 Jan 27;11(3):346. doi: 10.3390/plants11030346 (PMC8838583; doi:10.3390/plants11030346)
Supplement: Supplementary file 1 [file plants-11-00346-s001.zip › Supplementary File S1.pdf]

Supplementary File S1

- NMR spectrum of kiwifruit
- Chromatographic profiles obtained in negative ionization mode by UPLC-ESI-MS, with a method optimized for small molecule (**A**) and a standard method (**B**). The numbers in black represent the putatively annotated metabolites, described in Table 3. The numbers in red are the m/z values of abundant and unidentified metabolites.
- Table of relative contents of quinic acid

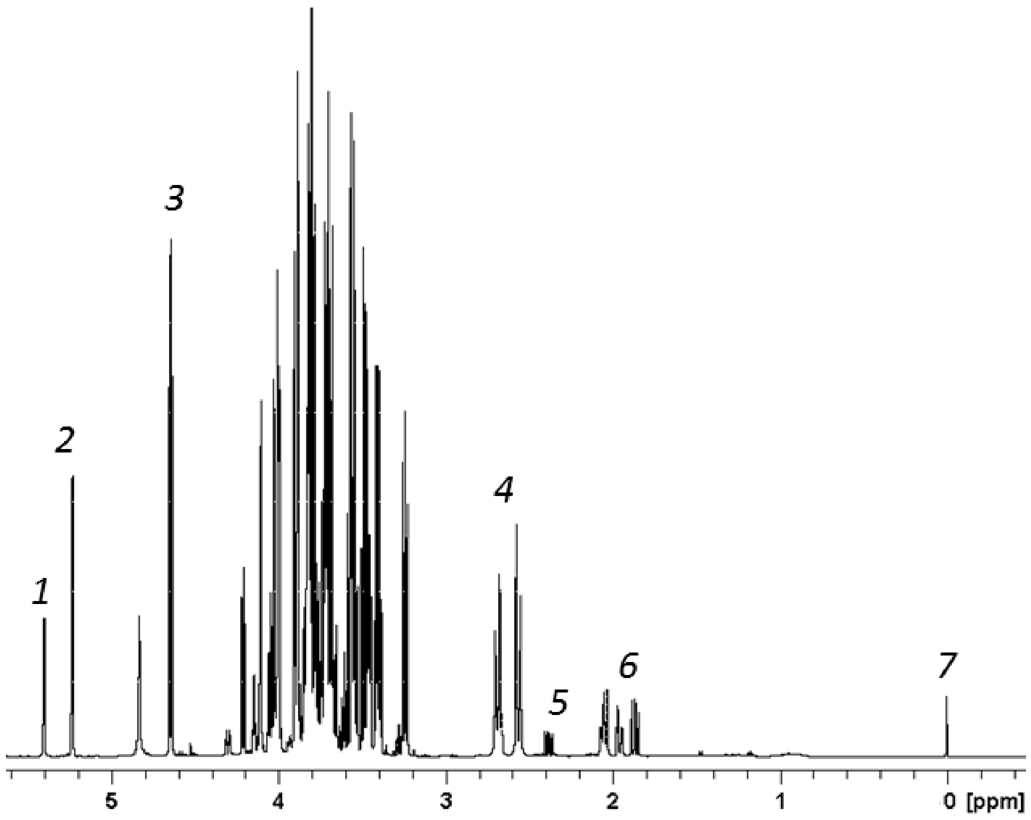

Representative ^{1}H NMR spectrum of Kiwi Hayward aqueous extract at 25°C. The assignment of selected peaks is reported: 1: sucrose; 2: α-glucose; 3: β-glucose; 4: citric acid; 5: malic acid; 6: quinic acid; 7: DSS-d6. The NMR-based quantification has been done for the most abundant metabolites that are sugars, and reported in milligrams of metabolites/ 100 grams of homogenized fruit powder. Sucrose: 1030 mg/100 g; Glucose: 3594 mg/100 g; Fructose: 3355 mg/100 g.

Carrot

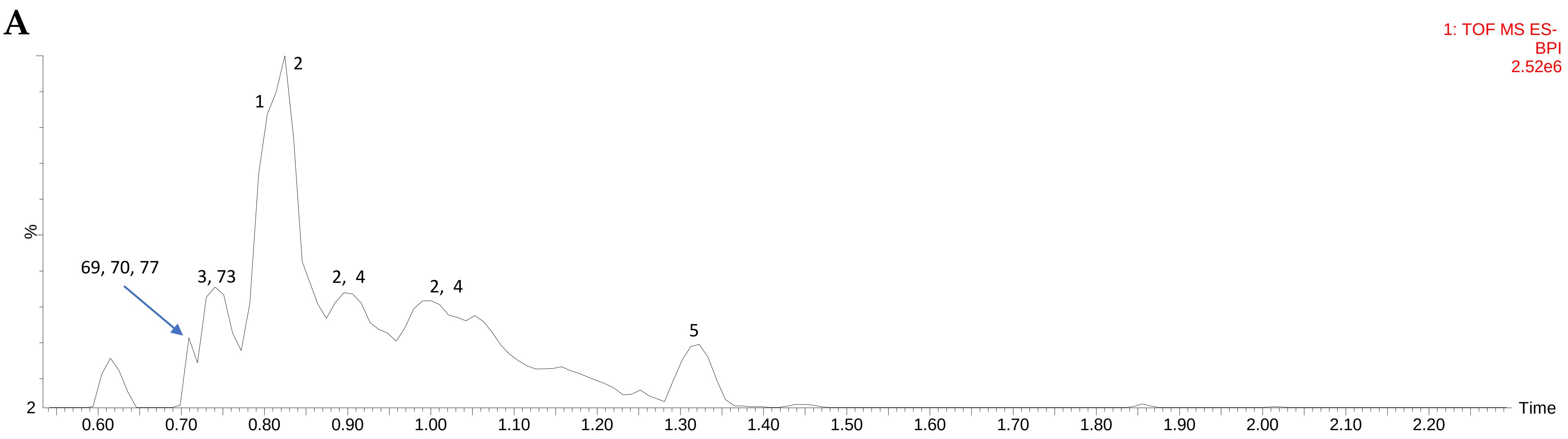
A
1: TOF MS ES-
BPI
2.52e6
%
69, 70, 77
3, 73
1
2
2, 4
2, 4
5
2
Time
0.60
0.70
0.80
0.90
1.00
1.10
1.20
1.30
1.40
1.50
1.60
1.70
1.80
1.90
2.00
2.10
2.20

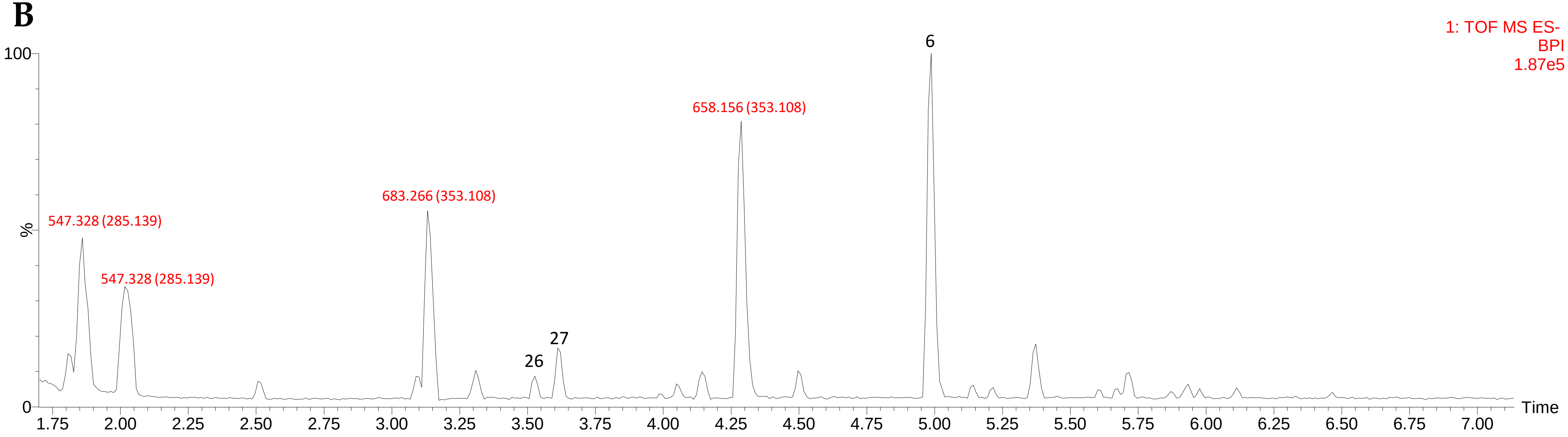
B
1: TOF MS ES-
BPI
1.87e5
100
%
0
547.328 (285.139)
547.328 (285.139)
683.266 (353.108)
26
27
658.156 (353.108)
6
Time
1.75
2.00
2.25
2.50
2.75
3.00
3.25
3.50
3.75
4.00
4.25
4.50
4.75
5.00
5.25
5.50
5.75
6.00
6.25
6.50
6.75
7.00

Cucumber

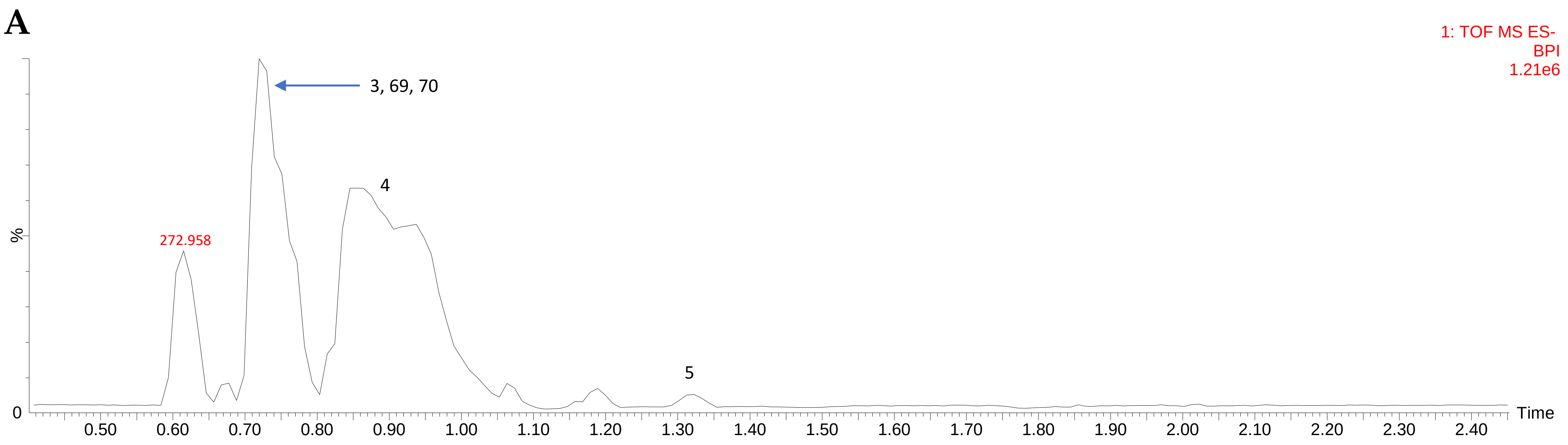

A
1: TOF MS ES-
BPI
1.21e6
3, 69, 70
4
272.958
5
%
0
Time
0.50
0.60
0.70
0.80
0.90
1.00
1.10
1.20
1.30
1.40
1.50
1.60
1.70
1.80
1.90
2.00
2.10
2.20
2.30
2.40

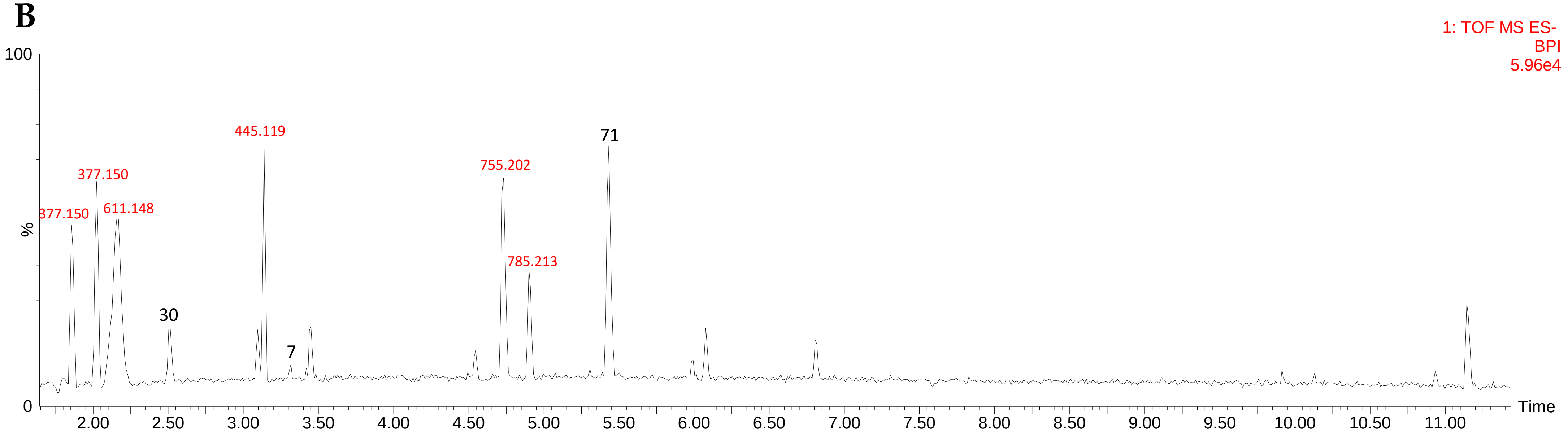

B
1: TOF MS ES-
BPI
5.96e4
100
%
0
445.119
71
377.150
755.202
377.150
611.148
785.213
30
7
Time
2.00
2.50
3.00
3.50
4.00
4.50
5.00
5.50
6.00
6.50
7.00
7.50
8.00
8.50
9.00
9.50
10.00
10.50
11.00

Cherry cv. Black Star

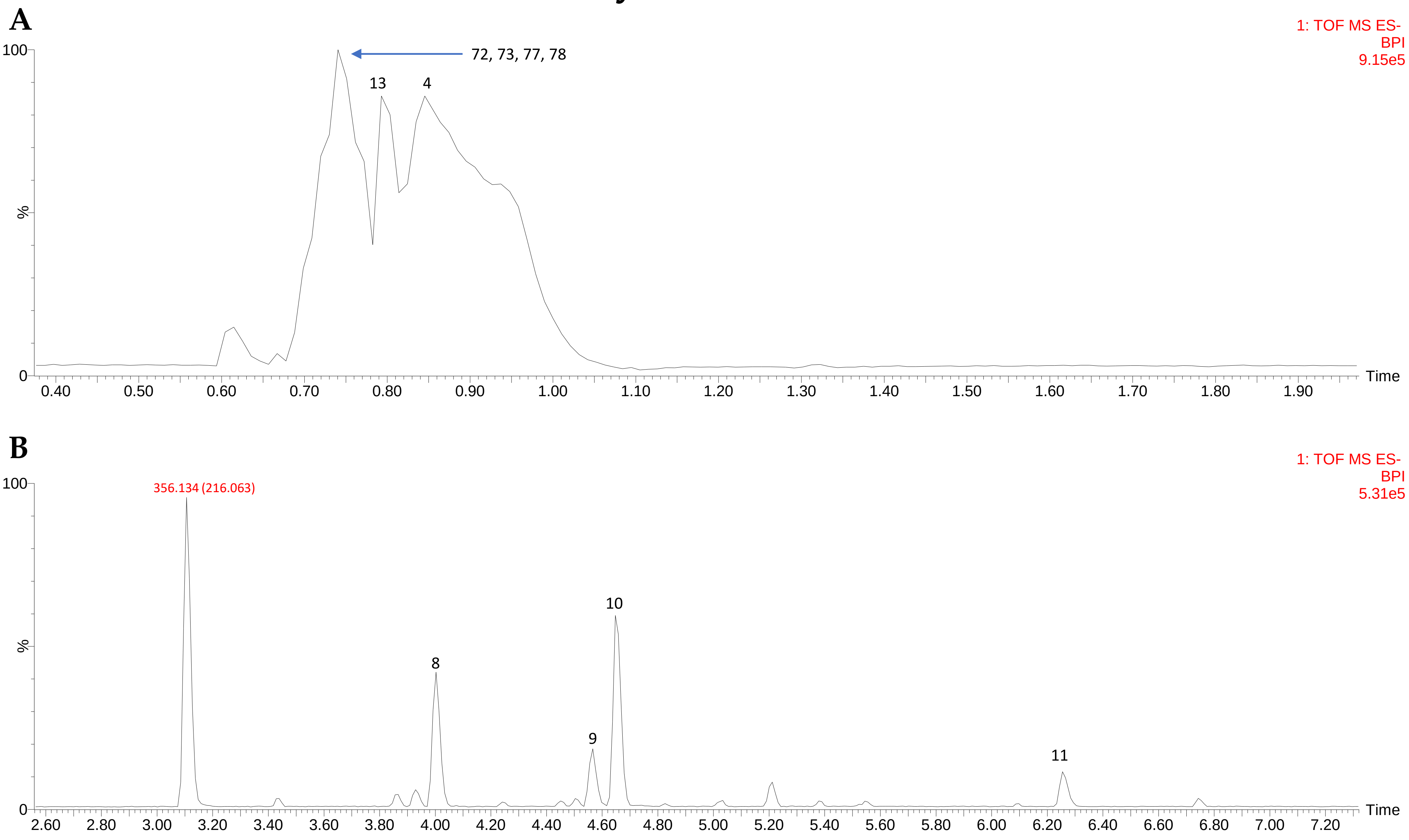

Cherry cv. Roana

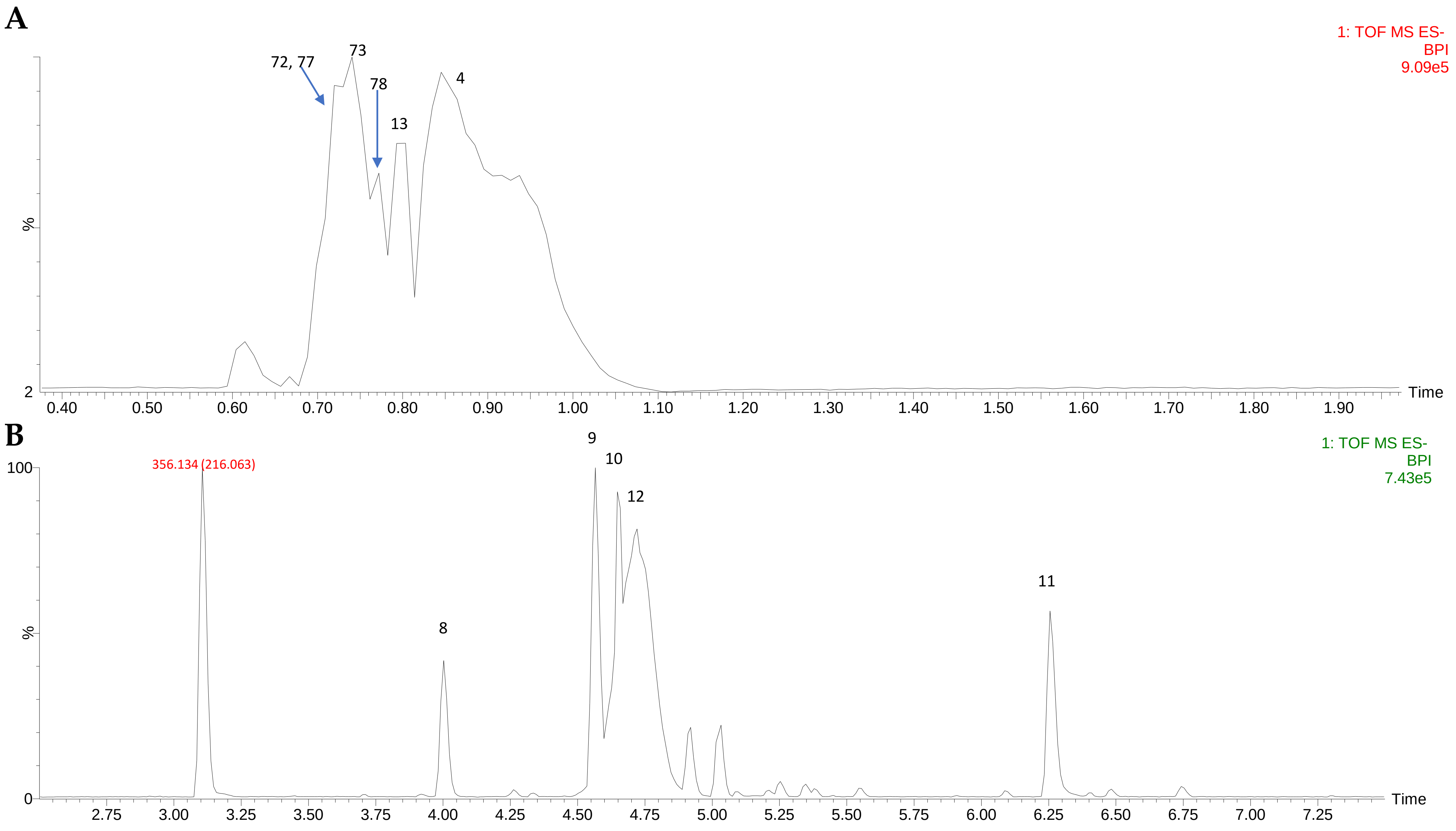

Onion

A

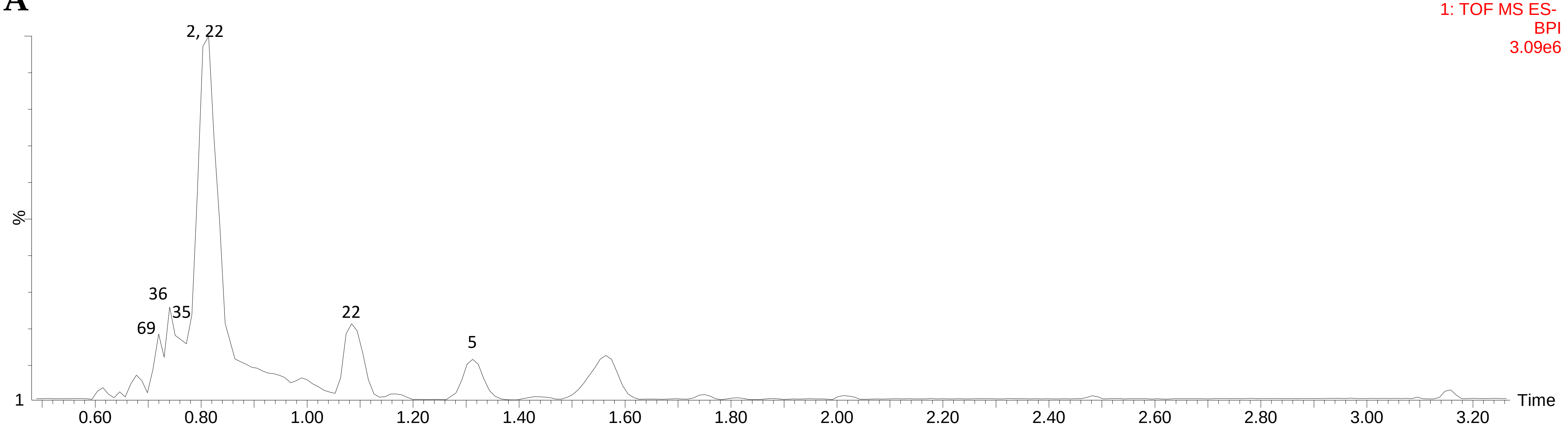
1: TOF MS ES-
BPI
3.09e6
2, 22
36
35
69
22
5
%
1
Time
0.60
0.80
1.00
1.20
1.40
1.60
1.80
2.00
2.20
2.40
2.60
2.80
3.00
3.20

B

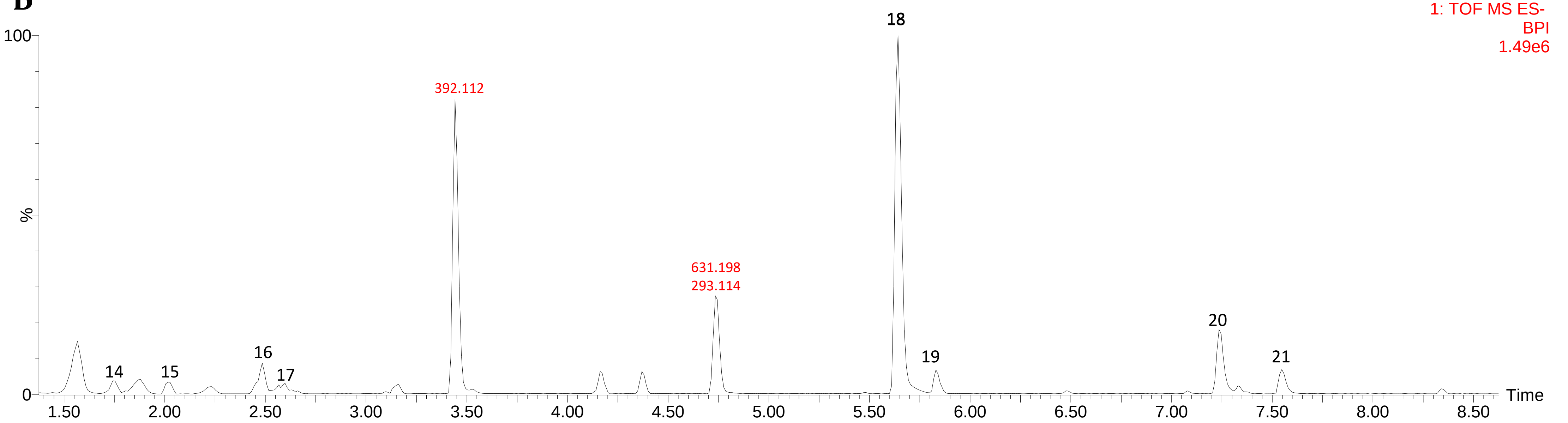
1: TOF MS ES-
BPI
1.49e6
18
392.112
631.198
293.114
14
15
16
17
19
20
21
100
%
0
Time
1.50
2.00
2.50
3.00
3.50
4.00
4.50
5.00
5.50
6.00
6.50
7.00
7.50
8.00
8.50

Shallot

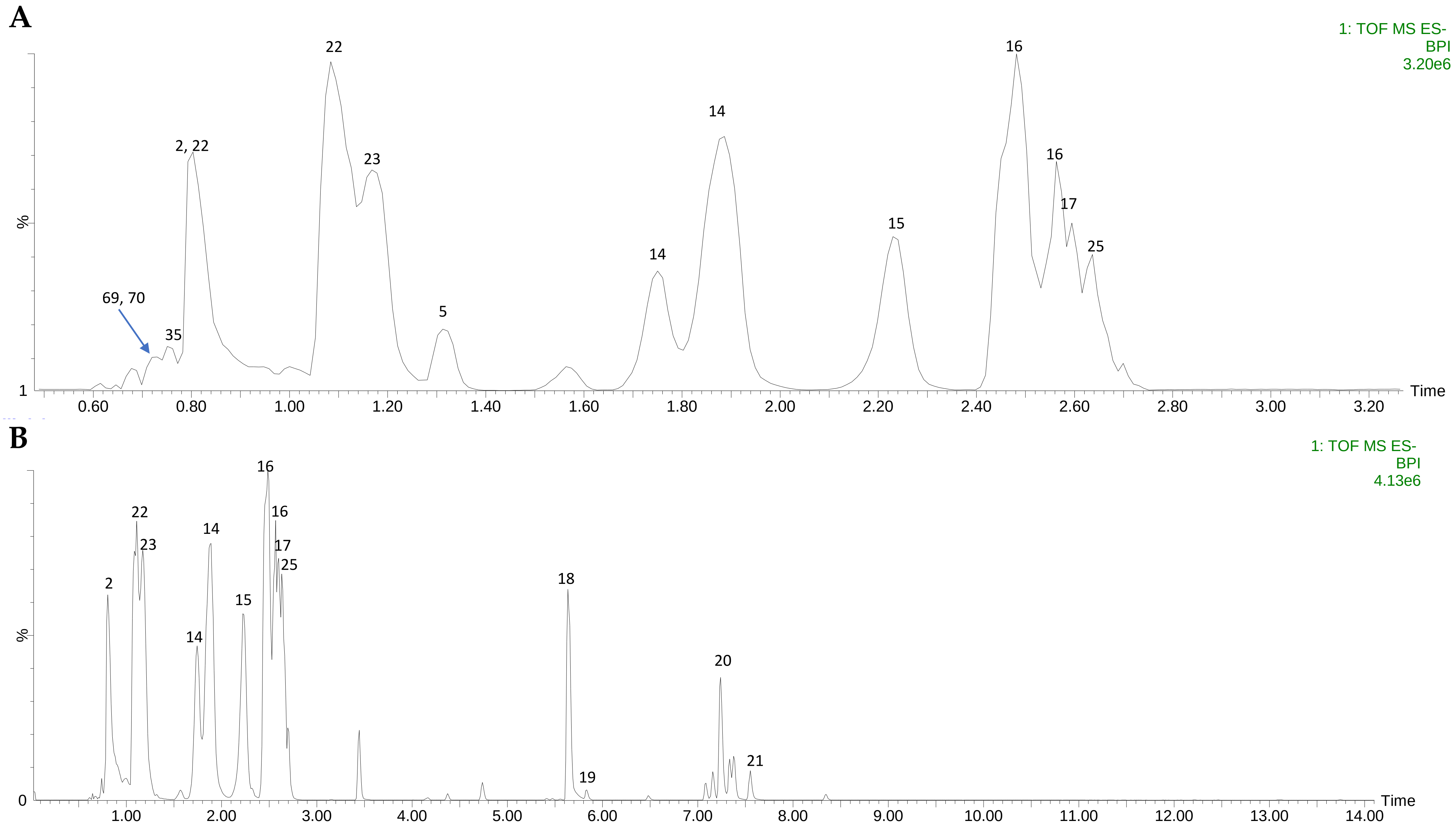
A
1: TOF MS ES-
BPI
3.20e6
%
Time
69, 70
35
2, 22
22
23
5
14
14
15
16
16
17
25
0.60
0.80
1.00
1.20
1.40
1.60
1.80
2.00
2.20
2.40
2.60
2.80
3.00
3.20
1
B
1: TOF MS ES-
BPI
4.13e6
%
Time
2
22
23
14
14
15
16
16
17
25
18
19
20
21
0
1.00
2.00
3.00
4.00
5.00
6.00
7.00
8.00
9.00
10.00
11.00
12.00
13.00
14.00

Fennel

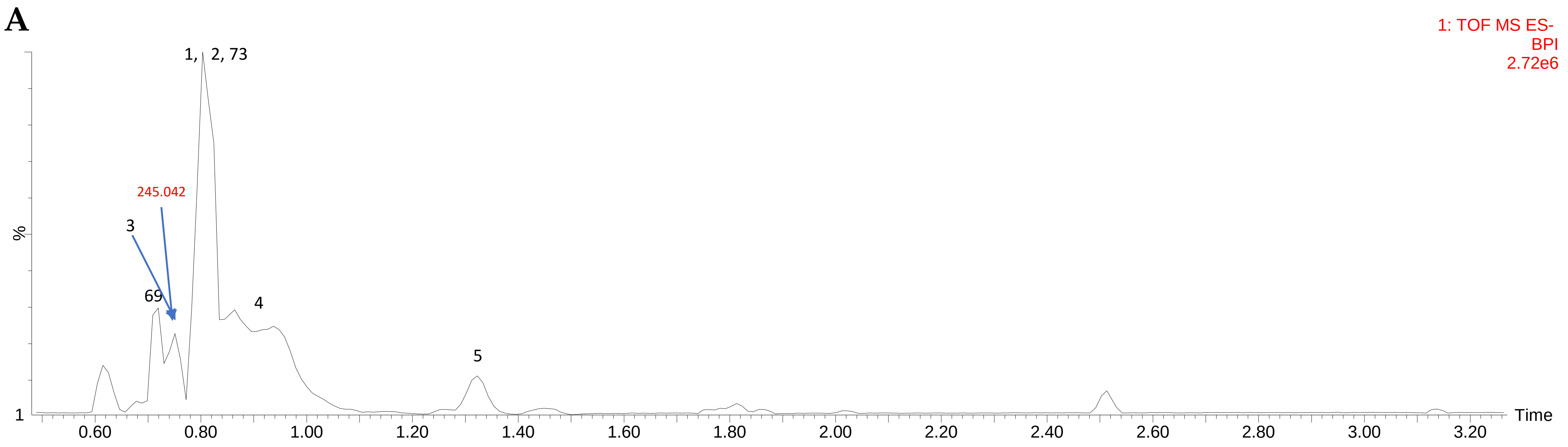

A
1: TOF MS ES-
BPI
2.72e6
1, 2, 73
245.042
3
69
4
5
%
1
Time
0.60
0.80
1.00
1.20
1.40
1.60
1.80
2.00
2.20
2.40
2.60
2.80
3.00
3.20

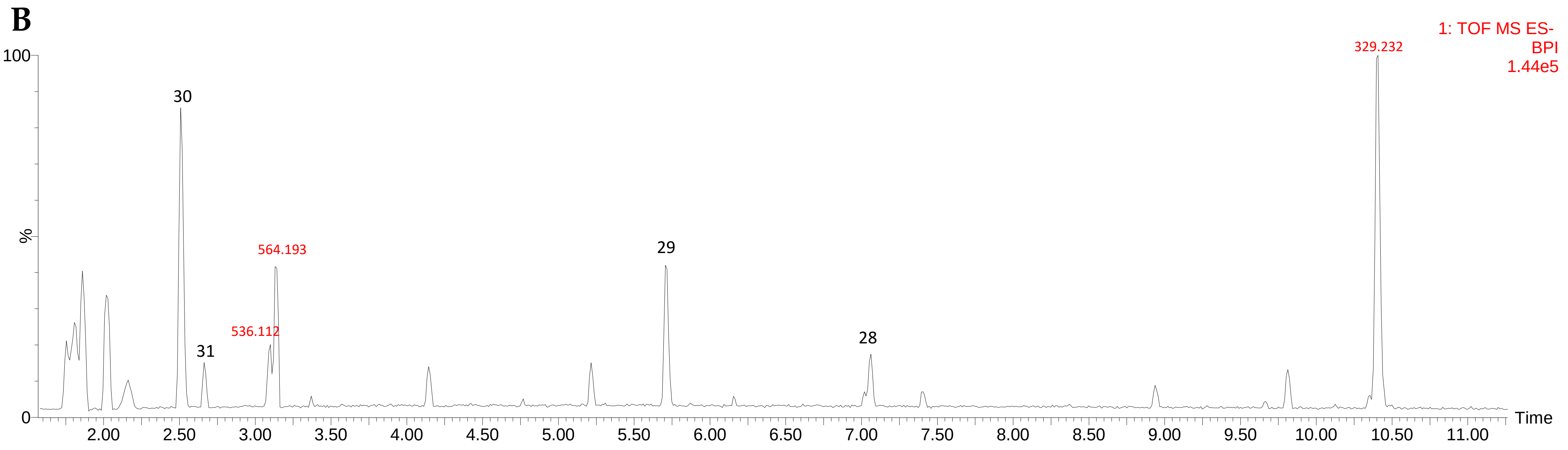

B
1: TOF MS ES-
BPI
1.44e5
329.232
100
30
%
564.193
29
536.112
31
28
0
Time
2.00
2.50
3.00
3.50
4.00
4.50
5.00
5.50
6.00
6.50
7.00
7.50
8.00
8.50
9.00
9.50
10.00
10.50
11.00

Kiwifruit

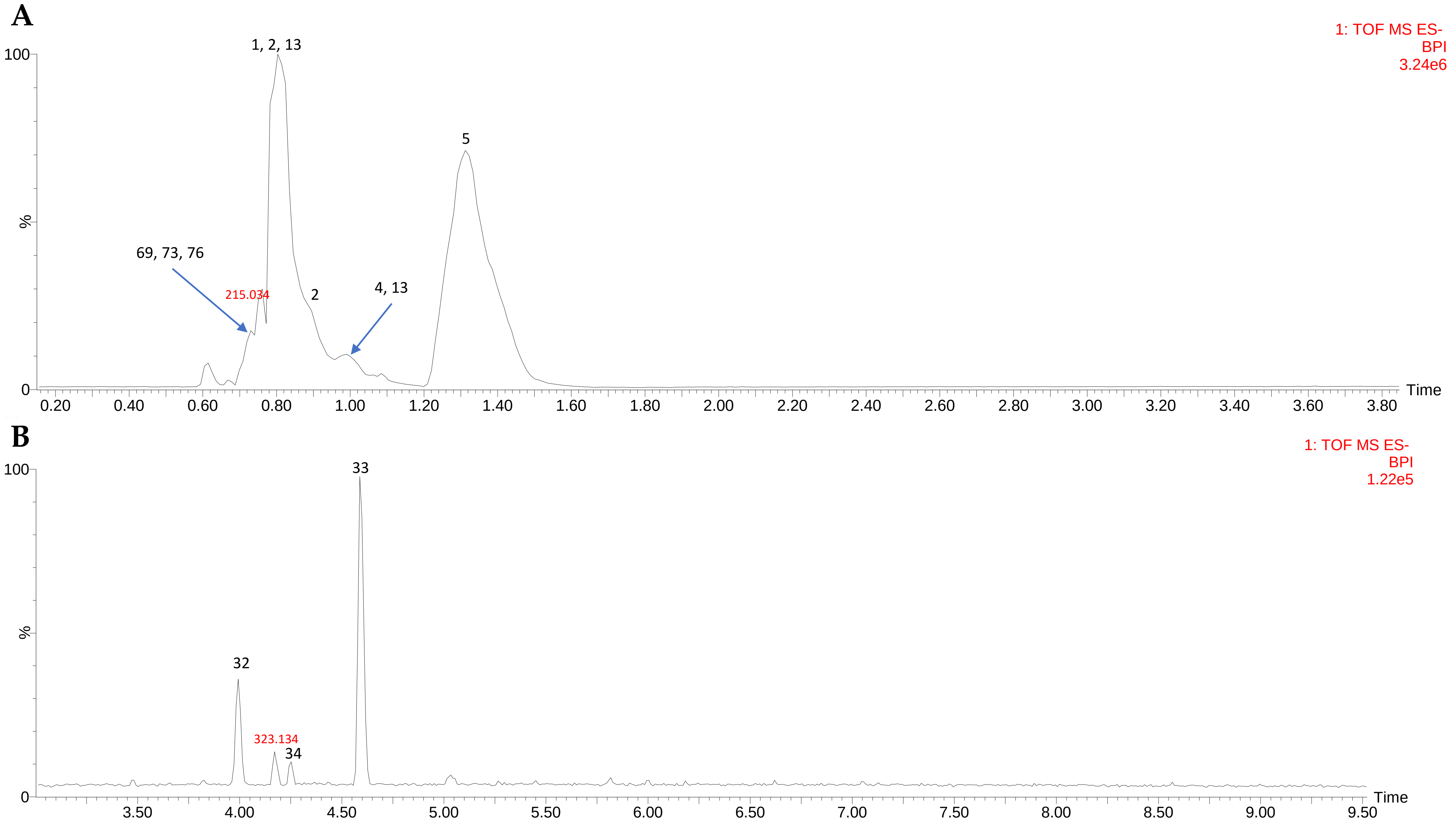
A
1: TOF MS ES-
BPI
3.24e6
100
%
0
1, 2, 13
5
69, 73, 76
215.034
2
4, 13
Time
0.20
0.40
0.60
0.80
1.00
1.20
1.40
1.60
1.80
2.00
2.20
2.40
2.60
2.80
3.00
3.20
3.40
3.60
3.80
B
1: TOF MS ES-
BPI
1.22e5
100
%
0
33
32
323.134
34
Time
3.50
4.00
4.50
5.00
5.50
6.00
6.50
7.00
7.50
8.00
8.50
9.00
9.50

Lettuce

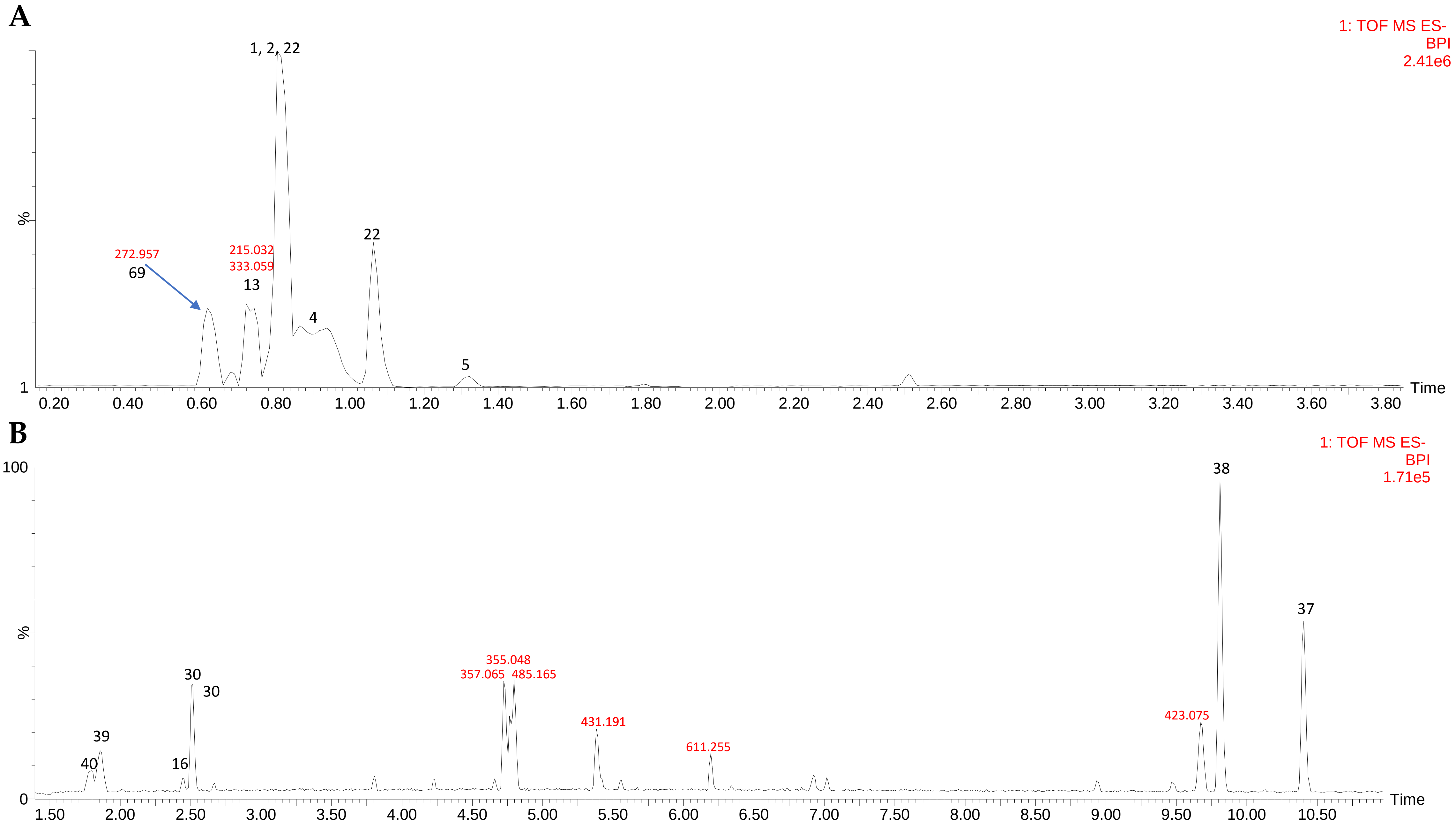
A
1: TOF MS ES-
BPI
2.41e6
1, 2, 22
272.957
69
215.032
333.059
13
4
22
5
%
1
Time
0.20
0.40
0.60
0.80
1.00
1.20
1.40
1.60
1.80
2.00
2.20
2.40
2.60
2.80
3.00
3.20
3.40
3.60
3.80
B
1: TOF MS ES-
BPI
1.71e5
100
%
0
38
37
30
30
39
40
16
355.048
357.065
485.165
431.191
611.255
423.075
Time
1.50
2.00
2.50
3.00
3.50
4.00
4.50
5.00
5.50
6.00
6.50
7.00
7.50
8.00
8.50
9.00
9.50
10.00
10.50

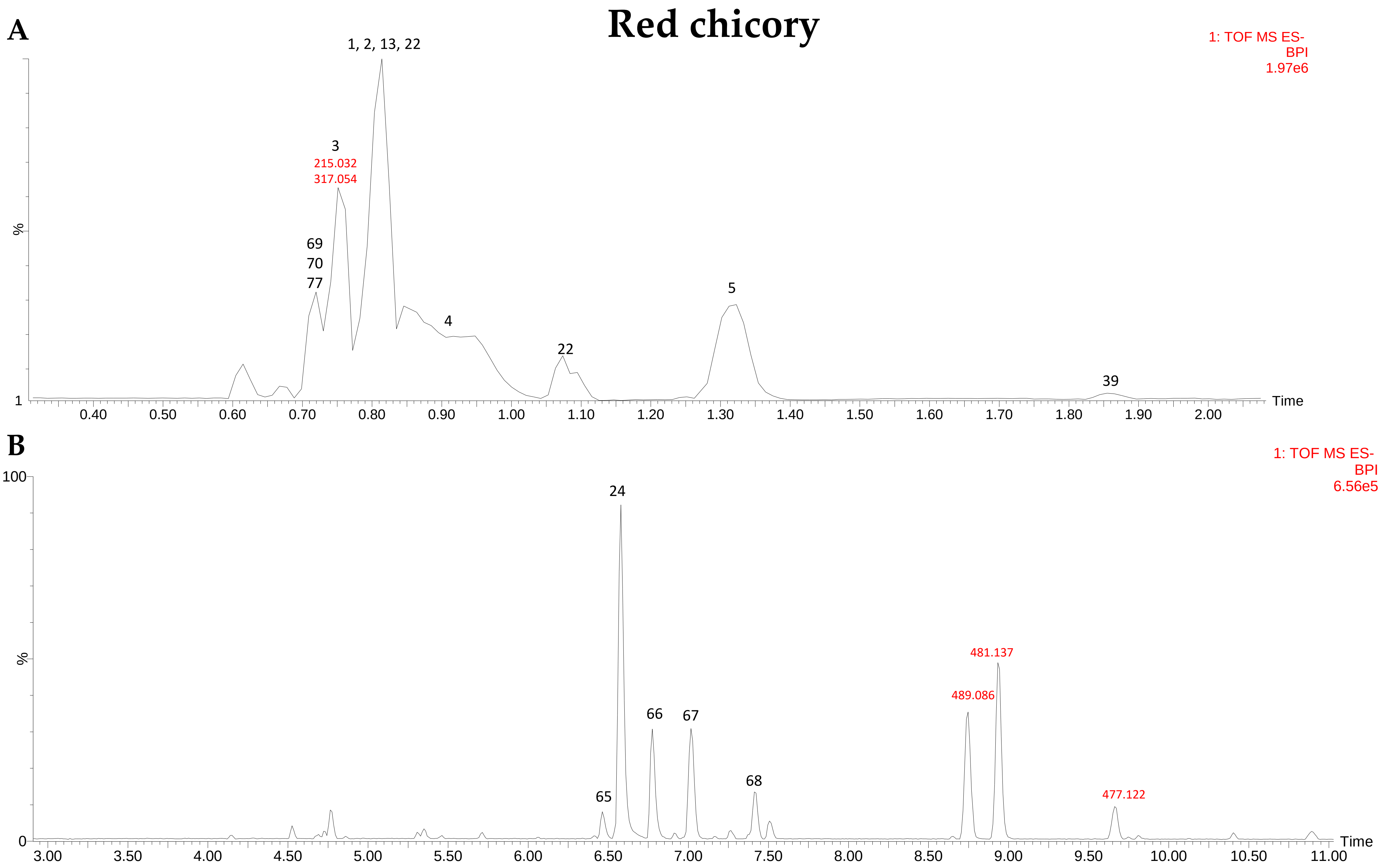

Red chicory
A
1: TOF MS ES-
BPI
1.97e6
1, 2, 13, 22
3
215.032
317.054
69
70
77
4
22
5
39
%
1
Time
0.40
0.50
0.60
0.70
0.80
0.90
1.00
1.10
1.20
1.30
1.40
1.50
1.60
1.70
1.80
1.90
2.00
B
1: TOF MS ES-
BPI
6.56e5
100
24
66
67
68
65
481.137
489.086
477.122
%
0
Time
3.00
3.50
4.00
4.50
5.00
5.50
6.00
6.50
7.00
7.50
8.00
8.50
9.00
9.50
10.00
10.50
11.00

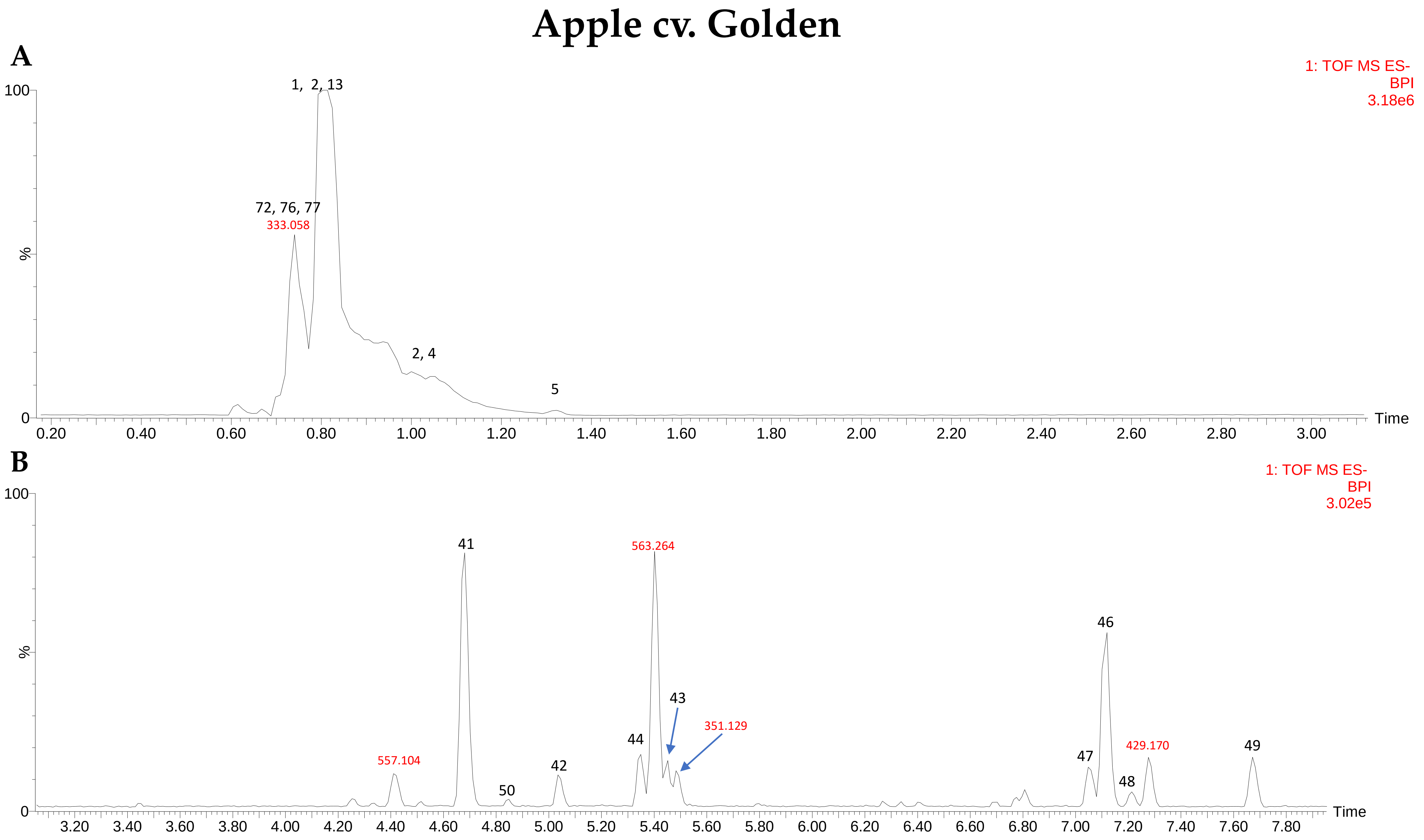

Apple cv. Golden
A
1: TOF MS ES-
BPI
3.18e6
1, 2, 13
72, 76, 77
333.058
2, 4
5
100
%
0
Time
0.20
0.40
0.60
0.80
1.00
1.20
1.40
1.60
1.80
2.00
2.20
2.40
2.60
2.80
3.00
B
1: TOF MS ES-
BPI
3.02e5
41
563.264
46
43
351.129
44
47
429.170
49
48
42
50
557.104
100
%
0
Time
3.20
3.40
3.60
3.80
4.00
4.20
4.40
4.60
4.80
5.00
5.20
5.40
5.60
5.80
6.00
6.20
6.40
6.60
6.80
7.00
7.20
7.40
7.60
7.80

Apple cv. Prussian

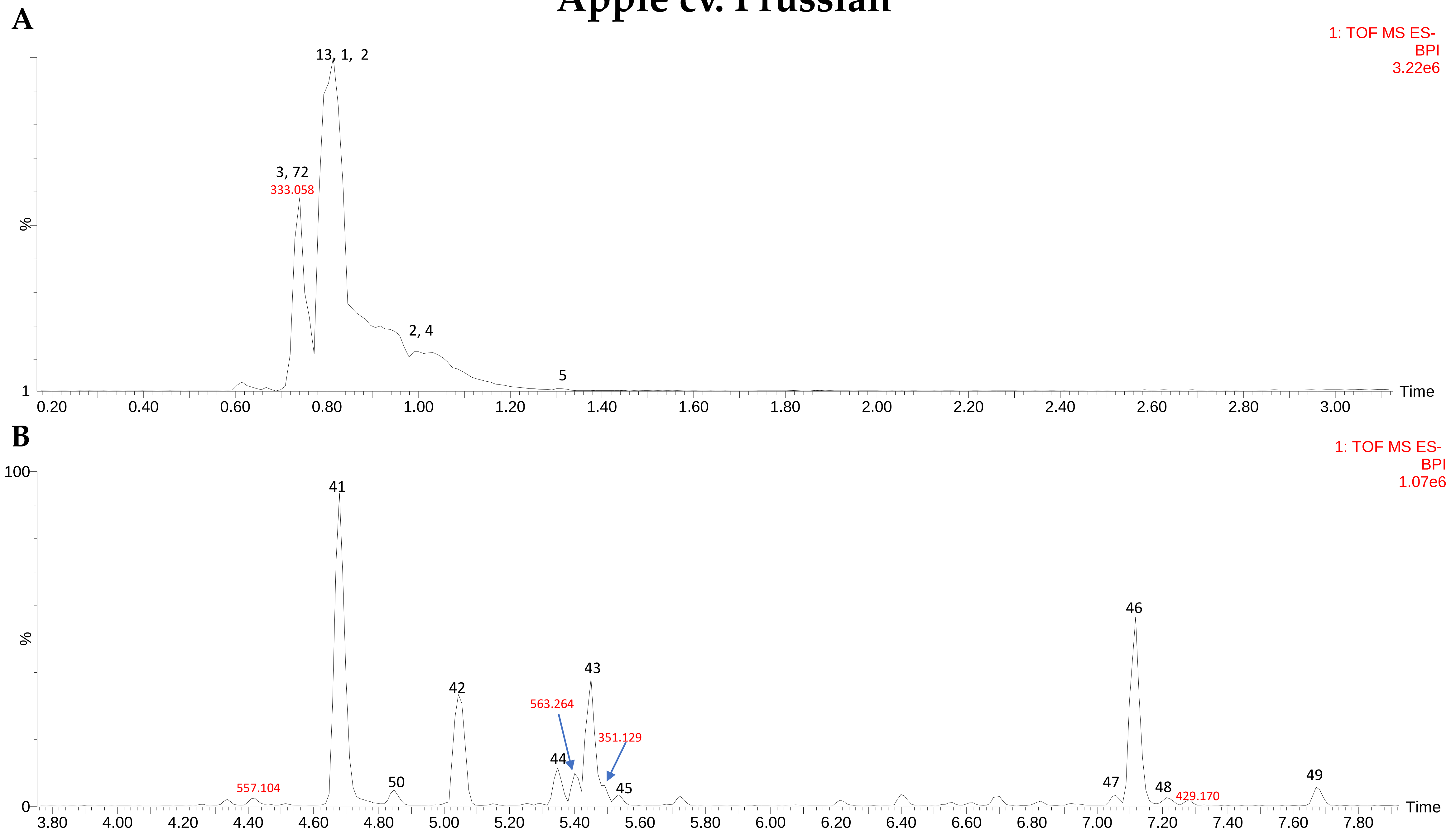

Pear

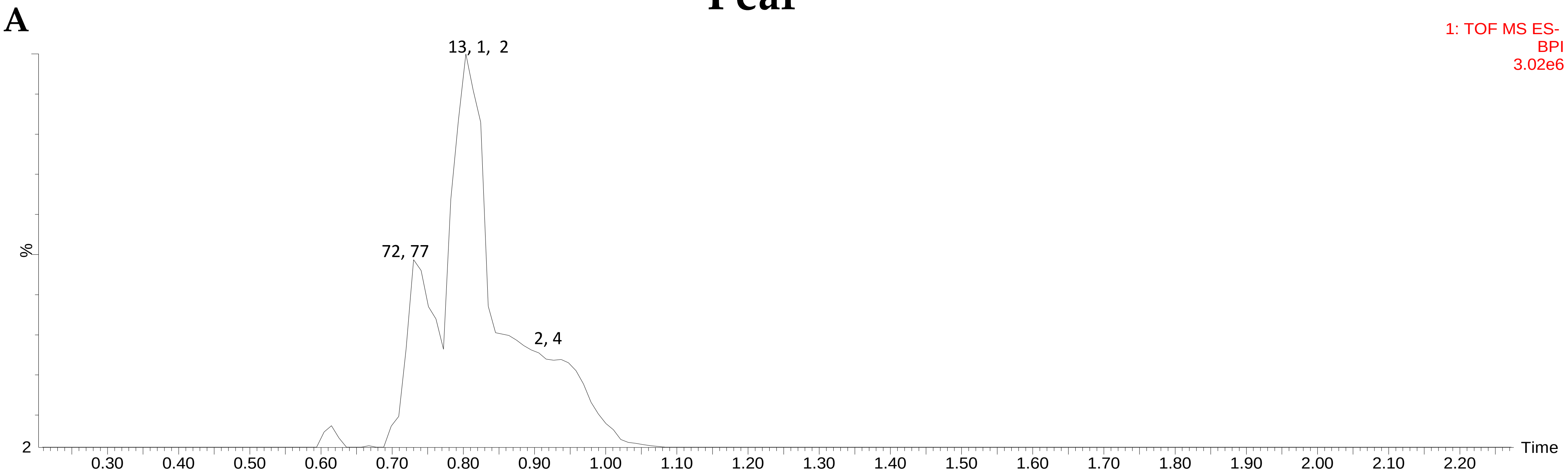
A
1: TOF MS ES-
BPI
3.02e6
13, 1, 2
72, 77
2, 4
%
2
Time
0.30
0.40
0.50
0.60
0.70
0.80
0.90
1.00
1.10
1.20
1.30
1.40
1.50
1.60
1.70
1.80
1.90
2.00
2.10
2.20

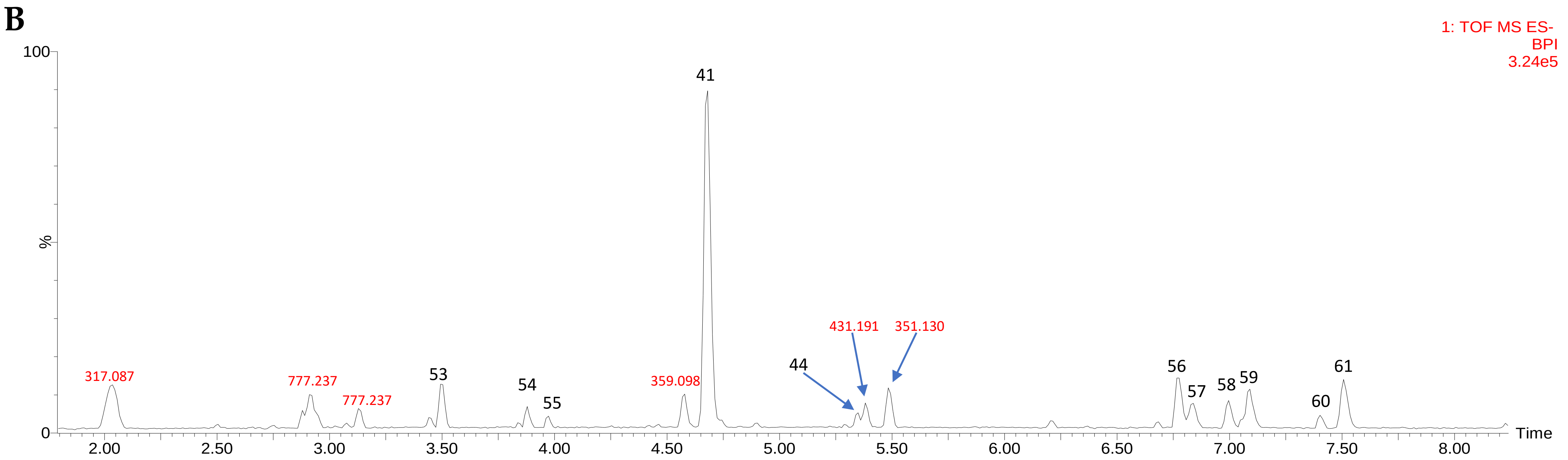
B
1: TOF MS ES-
BPI
3.24e5
100
%
0
41
317.087
777.237
777.237
53
54
55
359.098
44
431.191
351.130
56
57
58
59
60
61
Time
2.00
2.50
3.00
3.50
4.00
4.50
5.00
5.50
6.00
6.50
7.00
7.50
8.00

Nectarine

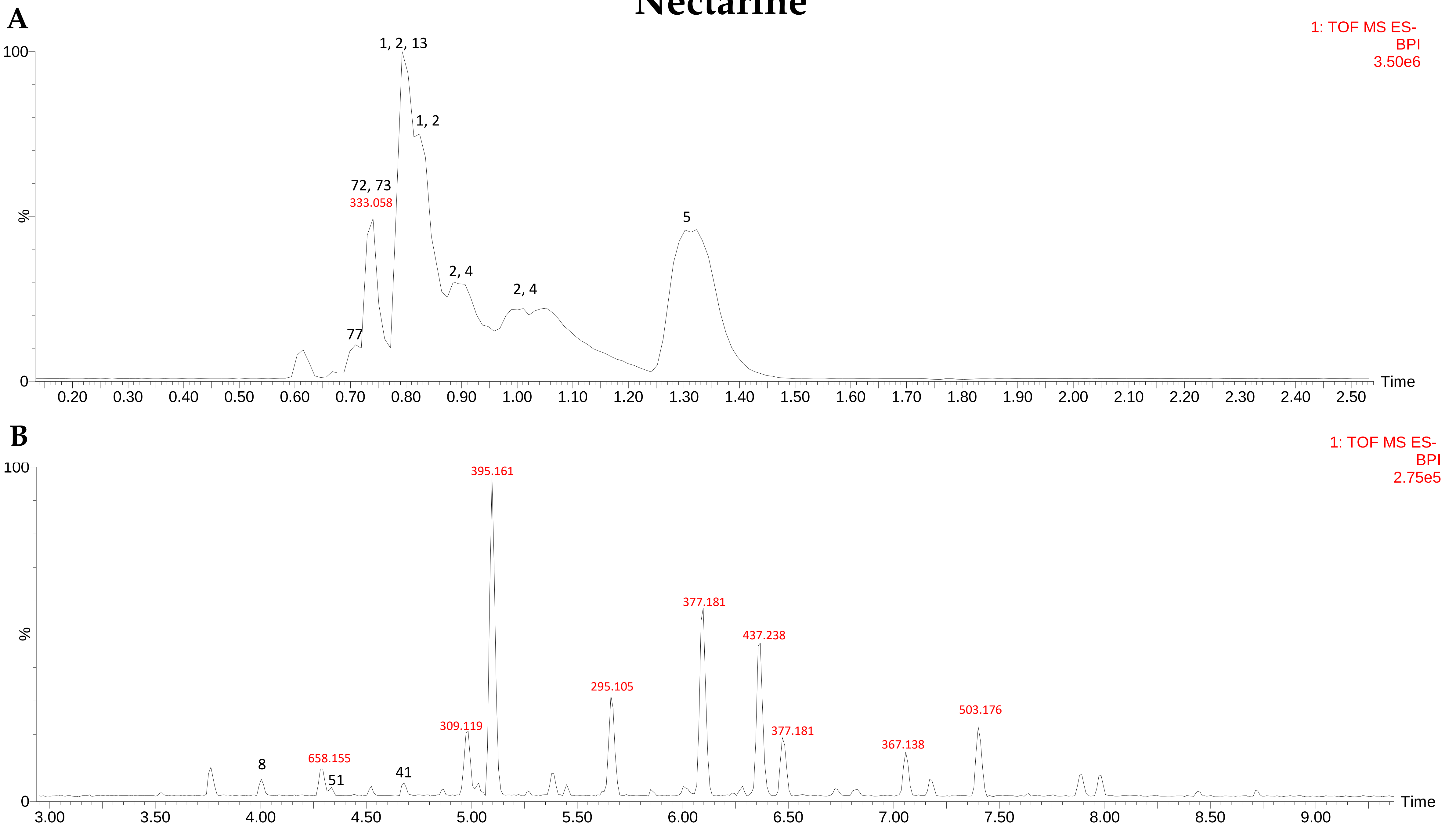
A
1: TOF MS ES-
BPI
3.50e6
100
%
0
1, 2, 13
1, 2
72, 73
333.058
77
2, 4
2, 4
5
Time
0.20 0.30 0.40 0.50 0.60 0.70 0.80 0.90 1.00 1.10 1.20 1.30 1.40 1.50 1.60 1.70 1.80 1.90 2.00 2.10 2.20 2.30 2.40 2.50
B
1: TOF MS ES-
BPI
2.75e5
100
%
0
395.161
377.181
437.238
295.105
309.119
503.176
377.181
367.138
658.155
8
51
41
Time
3.00 3.50 4.00 4.50 5.00 5.50 6.00 6.50 7.00 7.50 8.00 8.50 9.00

Peach

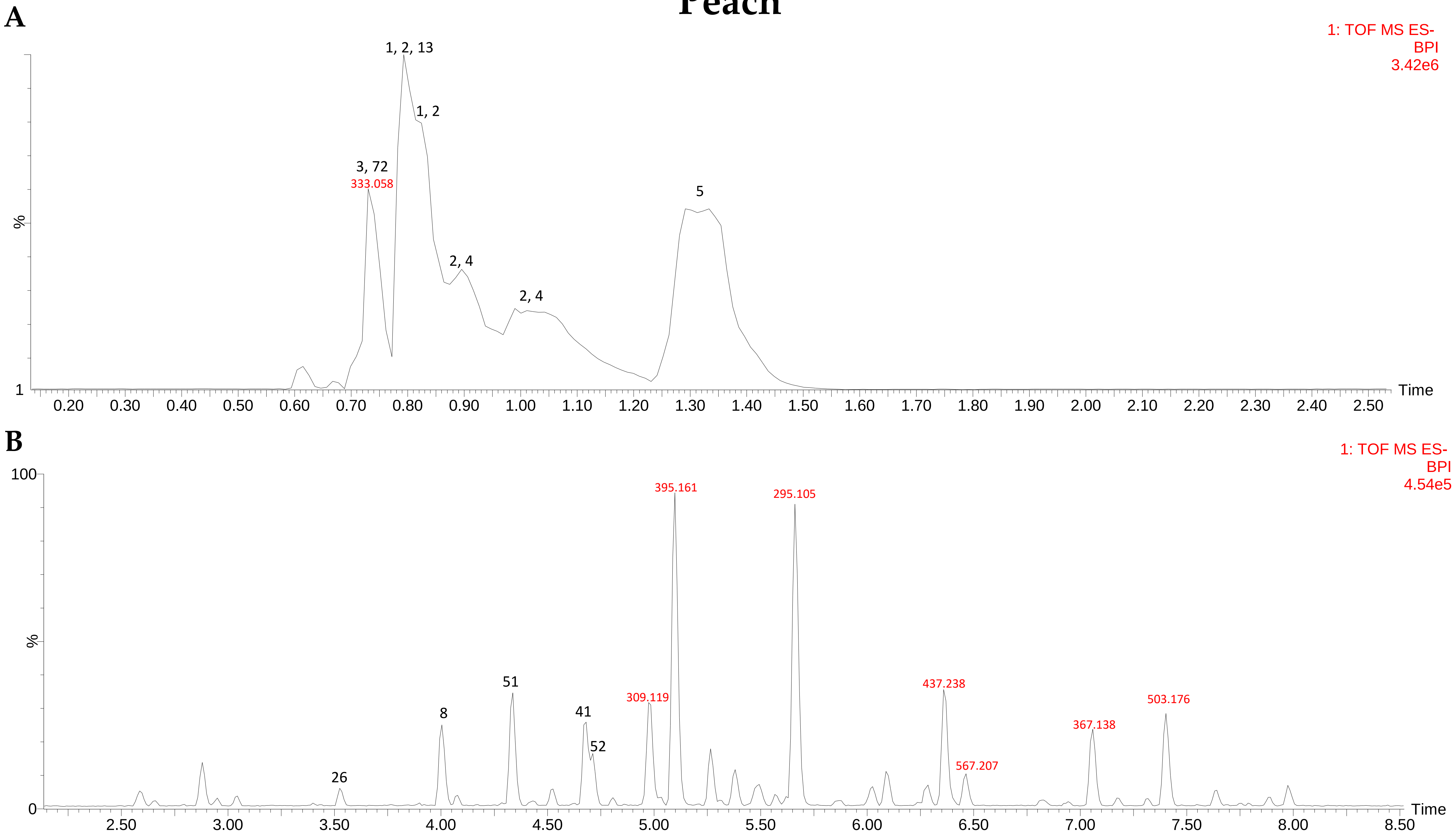
A
1: TOF MS ES-
BPI
3.42e6
1, 2, 13
1, 2
3, 72
333.058
5
2, 4
2, 4
%
1
Time
0.20 0.30 0.40 0.50 0.60 0.70 0.80 0.90 1.00 1.10 1.20 1.30 1.40 1.50 1.60 1.70 1.80 1.90 2.00 2.10 2.20 2.30 2.40 2.50
B
1: TOF MS ES-
BPI
4.54e5
100
395.161
295.105
%
51
8
41
52
309.119
437.238
503.176
367.138
567.207
26
0
Time
2.50 3.00 3.50 4.00 4.50 5.00 5.50 6.00 6.50 7.00 7.50 8.00 8.50

Ripe Bell Pepper

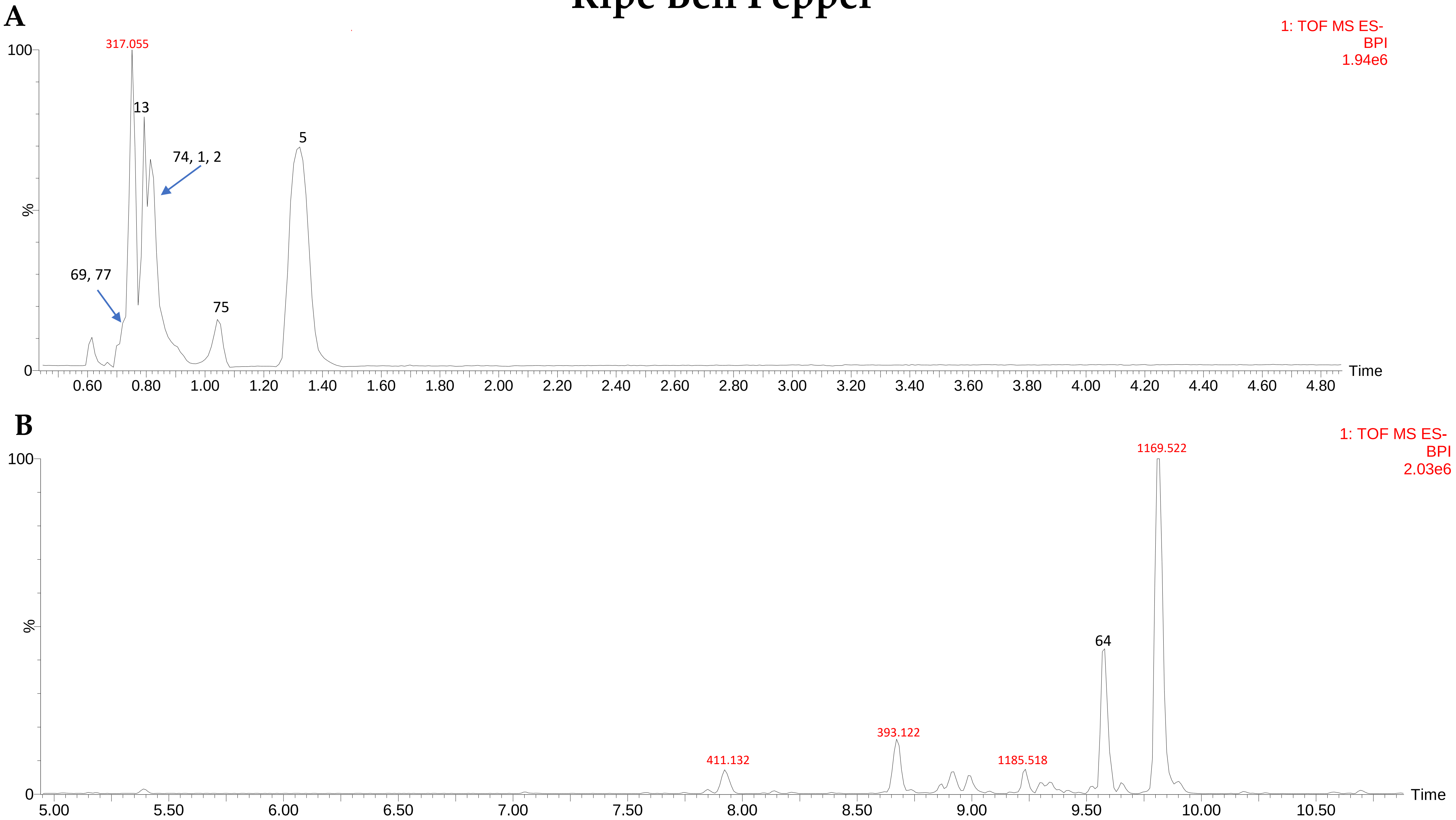

Unripe Bell Pepper

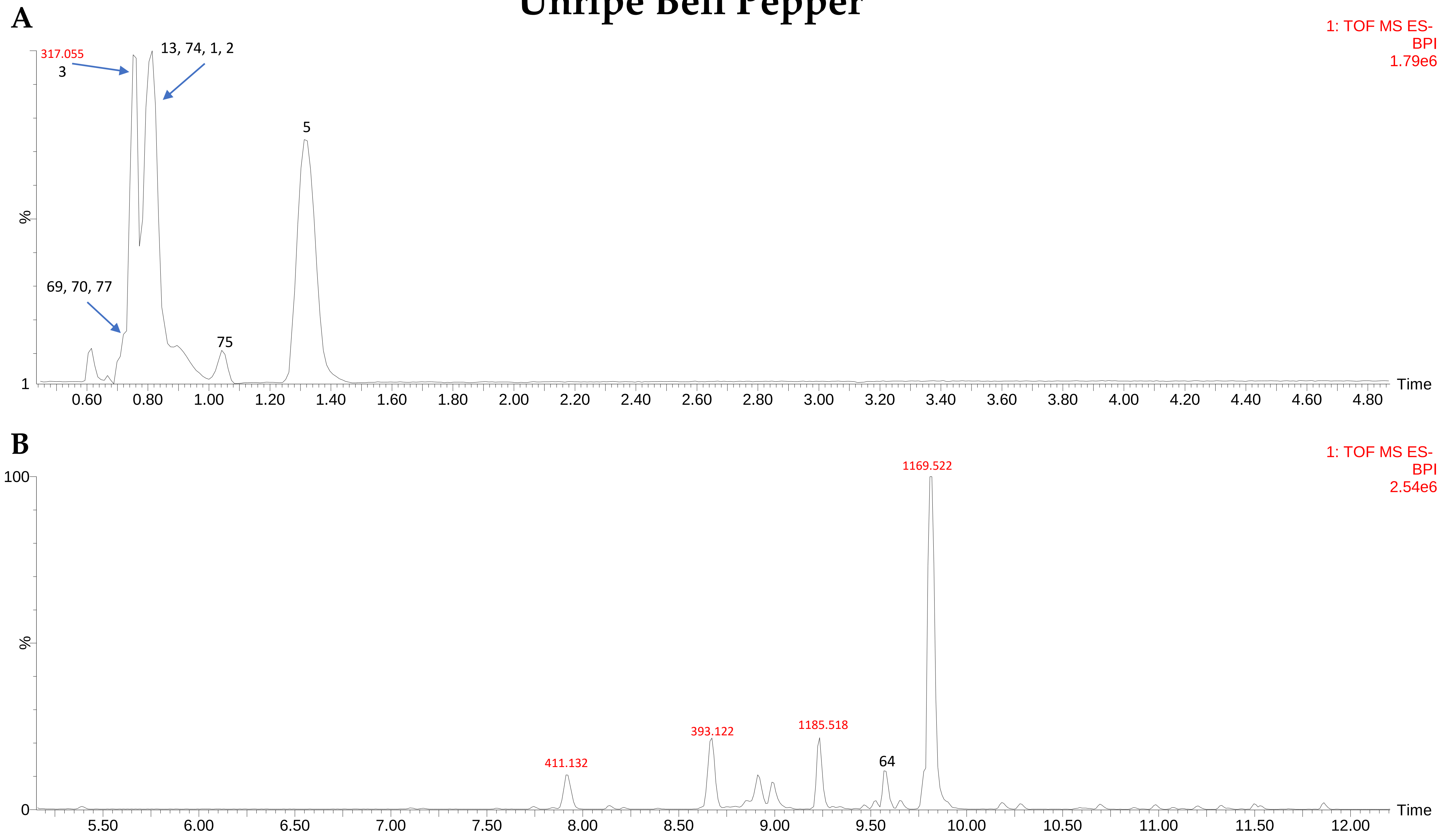

Tomato

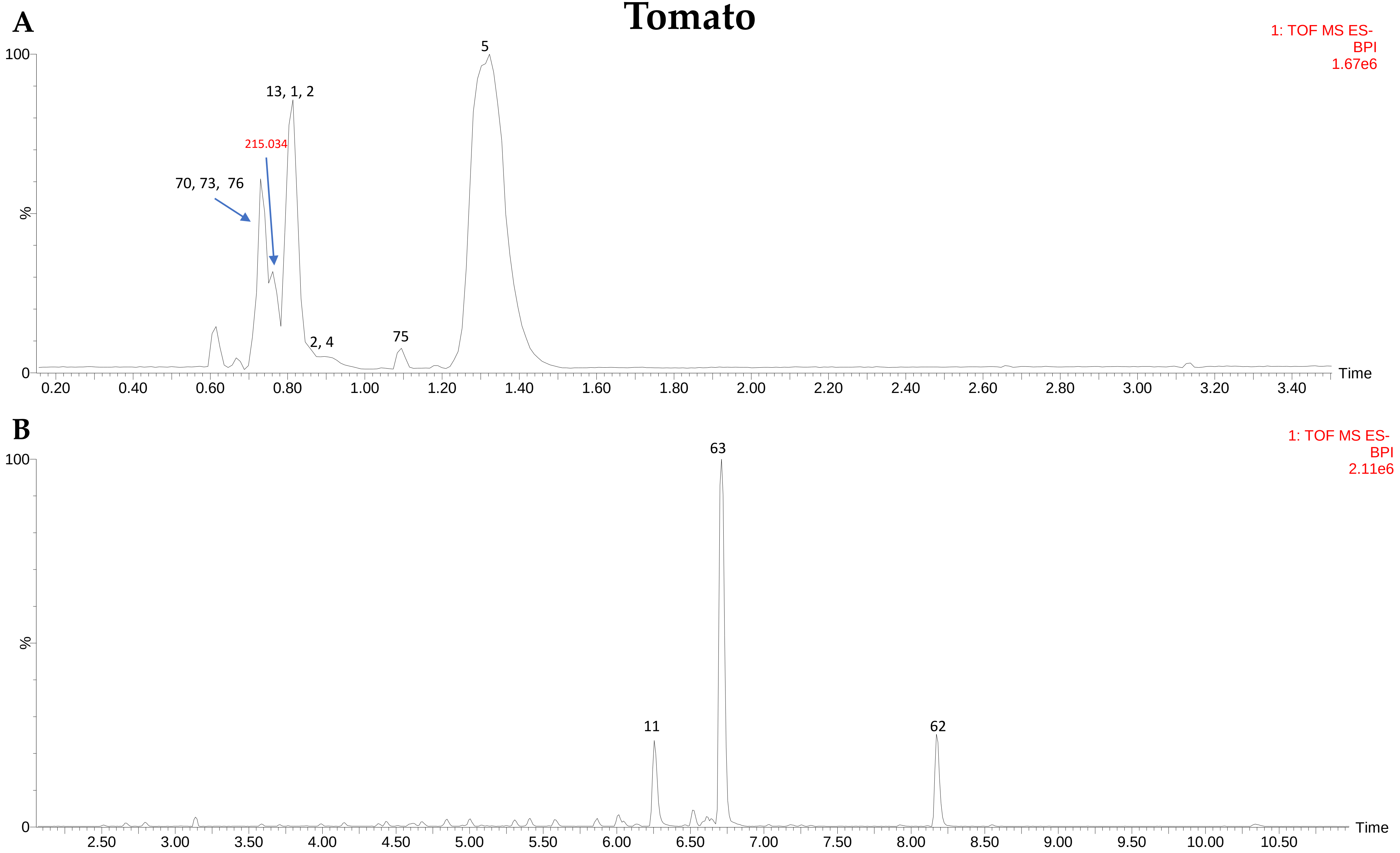
A
1: TOF MS ES-
BPI
1.67e6
100
%
0
5
13, 1, 2
215.034
70, 73, 76
2, 4
75
Time
0.20
0.40
0.60
0.80
1.00
1.20
1.40
1.60
1.80
2.00
2.20
2.40
2.60
2.80
3.00
3.20
3.40
B
1: TOF MS ES-
BPI
2.11e6
100
%
0
63
11
62
Time
2.50
3.00
3.50
4.00
4.50
5.00
5.50
6.00
6.50
7.00
7.50
8.00
8.50
9.00
9.50
10.00
10.50

Organ	Sample	Relative content of quinic acid (arbitrary units)	st.dev
Fruit	Kiwifruit	318.396	1.393
Fruit	Unripe green bell pepper	153.364	1.182
Fruit	Fully ripe red bell pepper	143.982	1.785
Fruit	Pear	114.408	1.375
Fruit	Peach	112.130	804
Fruit	Apple cv. Pom Prussian	101.022	1.220
Fruit	Nectarine	98.111	1.769
Fruit	Cherry cv. Black Star	69.258	436
Fruit	Cherry cv. Roana	61.892	787
Fruit	Tomato	59.250	3.765
Fruit	Apple cv. Golden	44.845	2.194
Leaves	Red chicory	31.561	1.646
Shoot	Fennel	25.108	1.458
Tap root	Carrot	16.400	549
Leaves	Lettuce	3.099	150
Bulb	Onion	641	83
Bulb	Shallot	610	26
Fruit	Cucumber	196	170

Comparison of relative content of quinic acid in all the sample, +/- standard deviation. The relative content is extrapolated from UPLC-MS raw data, is expressed in arbitrary units (i.e. area of the extracted peak of quinic acid, referred to the same amount of juice), and is the average of three independent samples. st.dev= standard deviation
