# Supplementary material for: Inhibition of Human Monoamine Oxidases A and B by Specialized Metabolites Present in Fresh Common Fruits and Vegetables
Source: Plants (Basel). 2022 Jan 27;11(3):346. doi: 10.3390/plants11030346 (PMC8838583; doi:10.3390/plants11030346)
Supplement: Supplementary file 1 [file plants-11-00346-s001.zip › Supplementary File S2.pdf]

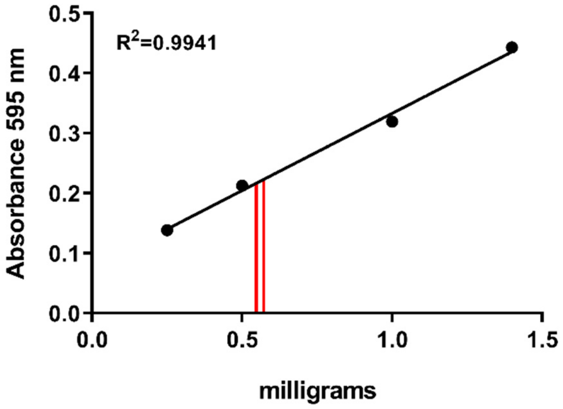

Compound	Protein concentration (mg/ml)	Optical Density (Entered)
	X	Y
Kiwifruit juice	0.548 ± 0.099	0.217
Neutral	0.573 ± 0.215	0.223

Table S1 and Figure S1. **Bradford Assay**
Absorbance was measured in kiwifruit and neutral solution by Bradford Assay. Values were evaluated by unpaired t-test and expressed as mean ± SD (n=4 per group).
